# The LMIT: Light-mediated minimally-invasive theranostics in oncology

**DOI:** 10.7150/thno.87783

**Published:** 2024-01-01

**Authors:** Yingwei Fan, Shuai Liu, Enze Gao, Rui Guo, Guozhao Dong, Yangxi Li, Tianxin Gao, Xiaoying Tang, Hongen Liao

**Affiliations:** 1 School of Medical Technology, Beijing Institute of Technology, Beijing, China, 100081.; 2 Dept. of Biomedical Engineering, School of Medicine, Tsinghua University, Beijing, China, 100084.

**Keywords:** minimally-invasive diagnosis and therapy, intelligent theranostics, intraoperative imaging, optical diagnosis and therapy, image-guided surgery

## Abstract

Minimally-invasive diagnosis and therapy have gradually become the trend and research hotspot of current medical applications. The integration of intraoperative diagnosis and treatment is a development important direction for real-time detection, minimally-invasive diagnosis and therapy to reduce mortality and improve the quality of life of patients, so called minimally-invasive theranostics (MIT). Light is an important theranostic tool for the treatment of cancerous tissues. Light-mediated minimally-invasive theranostics (LMIT) is a novel evolutionary technology that integrates diagnosis and therapeutics for the less invasive treatment of diseased tissues. Intelligent theranostics would promote precision surgery based on the optical characterization of cancerous tissues. Furthermore, MIT also requires the assistance of smart medical devices or robots. And, optical multimodality lay a solid foundation for intelligent MIT. In this review, we summarize the important state-of-the-arts of optical MIT or LMIT in oncology. Multimodal optical image-guided intelligent treatment is another focus. Intraoperative imaging and real-time analysis-guided optical treatment are also systemically discussed. Finally, the potential challenges and future perspectives of intelligent optical MIT are discussed.

## 1. Introduction

Cancer is a very difficult disease to cure and represents a group of diseases characterized by rapidly proliferating cells that can spread to other tissues in a process called metastasis. The complete resection of tumorous tissues by surgery or direct ablation is also challenging due to the difficulty in the identification of residual cancerous tissues. In recent decades, precision medicine has been the focus in the treatment of cancers 1. Light has been widely used in modern biomedical and clinical applications 2,3. Optical diagnosis, therapy are important methods for the treatment of cancerous tissue. Currently, with the rapid development of optical technology (OT), information technology (IT) and artifact intelligence (AI) 4, the intelligent integration of precision diagnosis and interventional therapeutics for minimally-invasive surgery (MIS) plays an important role in the treatment of soft tissue carcinomas, i.e., minimally-invasive theranostics (MIT). Currently, some minimally-invasive devices based on optical technology, so-called optical MIT devices or light-mediated MIT (LMIT) devices, are routinely researched and developed in the clinical applications.

During malignant tumor eliminating (such as multiform glioblastoma, GBM), the conventional therapeutic is surgical resection based on skilled surgeons' experience during an open operation 2. In the conventional treatment of tumors, surgical resection is also a primary and conventional means. Computer-assisted surgery 5,6 can provide a more convenient and intuitive view for the real-time resection of soft tissue sarcoma to increase the resection accuracy, maximize normal tissue and improve the success rate of surgical resection. The quantitative identification of tumors can also precisely guide surgery or other treatment methods. To identify and segment tumorous tissue, some biomedical technologies have been proposed to acquire real-time intraoperative data and guide surgical operations. Light-mediated methods 7,8, magnetic resonance imaging (MRI) 9, X-ray 10, computer tomography (CT) 11, ultrasound 12 and ultrasound theranostics 13, five-aminolevulinic acid (5-ALA) fluorescence 14, Raman spectroscopy 15, mass spectrometry 16, laser speckle contrast imaging (LSCI) 17, multimodality imaging 18, and terahertz technology 19 have been used in clinical trials. Optical diagnosis is an effective tool for identifying diseased tissue and normal tissues to guide precise surgical resection. Hyperspectral imaging 20-22, photoacoustic imaging (PAI) 23-26 and optical coherence tomography (OCT) 27-30 have been investigated in identifying tumors and normal tissues or guiding the surgical resection of lesions.

Currently, surgery is the main treatment method for tumorous tissues 31. Optical treatment as an effective treatment tool for tumorous tissues has gradually been investigated in clinical trials. MIT is a future direction in the treatment of diseased tissue. Laser ablation has been integrated into a theranostic system to diagnose and treat brain tumors 32,33. Laser ablation is used as a rapid diagnostic tool to identify tumorous tissues and guide treatment of cancers. Clinical trials need direct views of the tumorous area for surgeons to guide surgical implementation. Currently, some methods, such as laser ablation, photodynamic therapy (PDT) and photothermal treatment (PPT) 2, are also useful for the treatment of diseased tissues. Similarly, before intelligent treatment, precision diagnosis, including pathology 34, provides precise and therapeutic guidance. However, these technologies still have some disadvantages, and some of them have low-resolution, low-intelligence and low-speed imaging such that they cannot achieve intraoperative real-time intelligent identification of tumorous tissues, junction zones and normal tissues. The integration of diagnosis and therapeutics, i.e., theranostics, is a novel development trend in clinical application. The technology meets the requirements of interventional operation and minimally-invasive treatment 35. Combined with optical biomarkers, optical imaging and diagnosis could be enhanced and provide a biomedical detection method for contrast enhancement. However, suffering from intraoperative imaging, real-time and automatic diagnosis, and effective navigation, theranostics is still not realized intelligently and effectively in current clinical trials. Medical optical technology, including optical diagnosis, and treatment technologies, provides effective and precise diagnosis and therapeutic information. Overall, light-mediated diagnosis and therapy may be a potential high-precision solution for the treatment of soft tissue carcinoma for future intelligent MIT.

**Figure 1:**
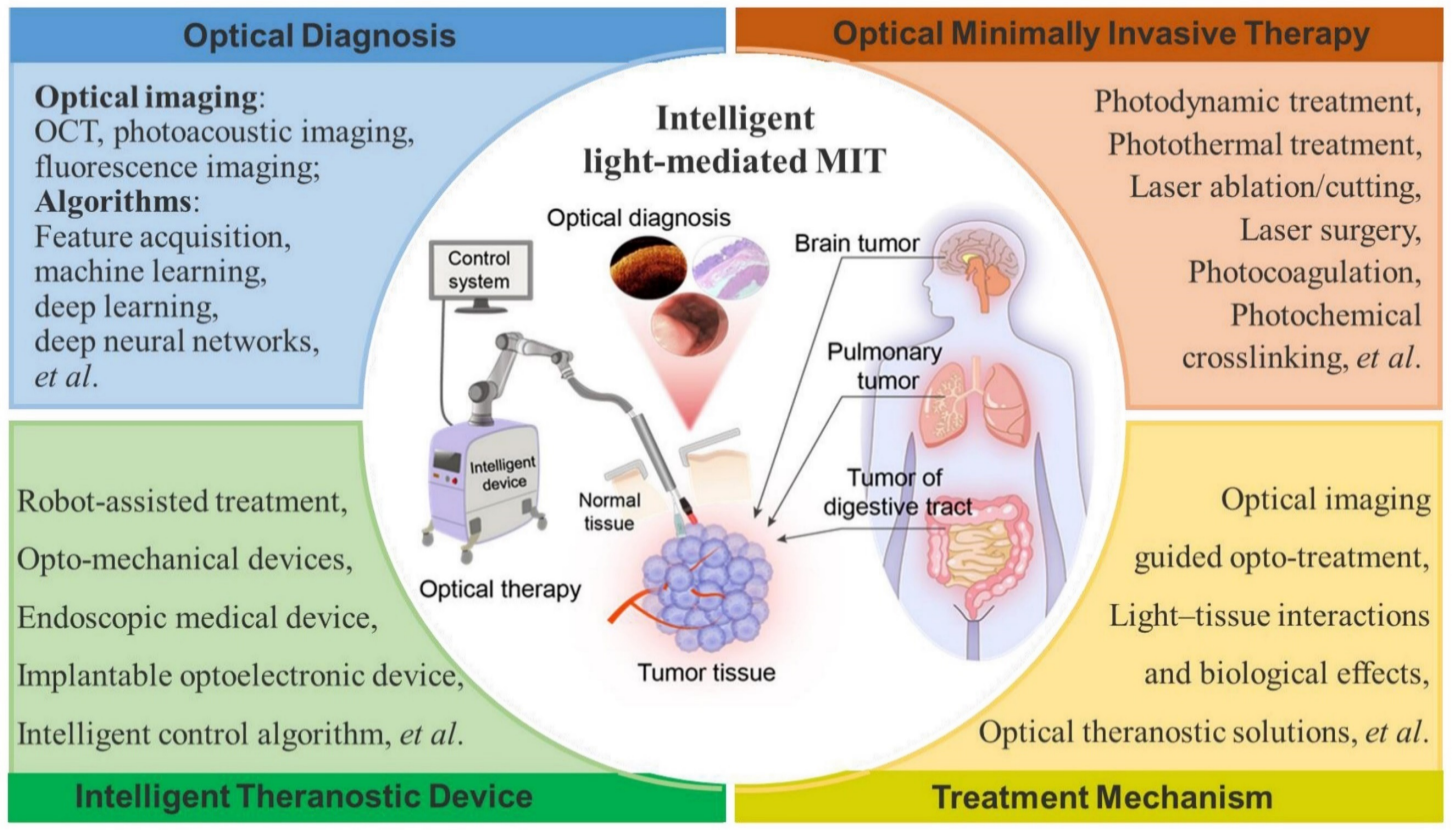
Light-mediated minimally-invasive theranostics (LMIT) or optical minimally-invasive theranostics (OMIT).

In this paper, we summarize the recent advances in optical diagnosis, therapy, and intelligent MIT (**Figure** 1) and highlight the future perspectives of intelligent MIT. Intraoperative imaging-guided treatment provides great convenience in the treatment of cancerous tissues. Multimodal image-guided treatment mediated by optical technology is also reviewed and discussed. Intelligent light-medicated MIT could be effectively employed in clinical applications in the future.

## 2. Optical diagnosis and therapy

Intraoperative diagnosis is important for MIS. Optical- imaging biomarkers 36 provide sufficient information in clinical workflows for the noninvasive detection and characterization of disease states. The precision of the clinical diagnosis directly determines the treatment effect. Furthermore, MIS requires real-time information and imaging to support clinical decision-making. **Table 1** lists the main topics to be addressed in this review illustrating the key features and current and future research trends in the field of light-mediated diagnosis, treatment, and theranostics.

### 2.1 Optical diagnosis

Optical diagnosis tools, such as OCT, confocal microscopy and photoacoustic imaging, have been widely used in the research on intraoperative surgical diagnosis. Label-free imaging 34,37 is a very potentially outstanding diagnostic method. Hence, optical imaging is a very powerful intraoperative tool in guiding oncologic surgeons in radical resection to achieve optimal clinical results.

**Figure 2:**
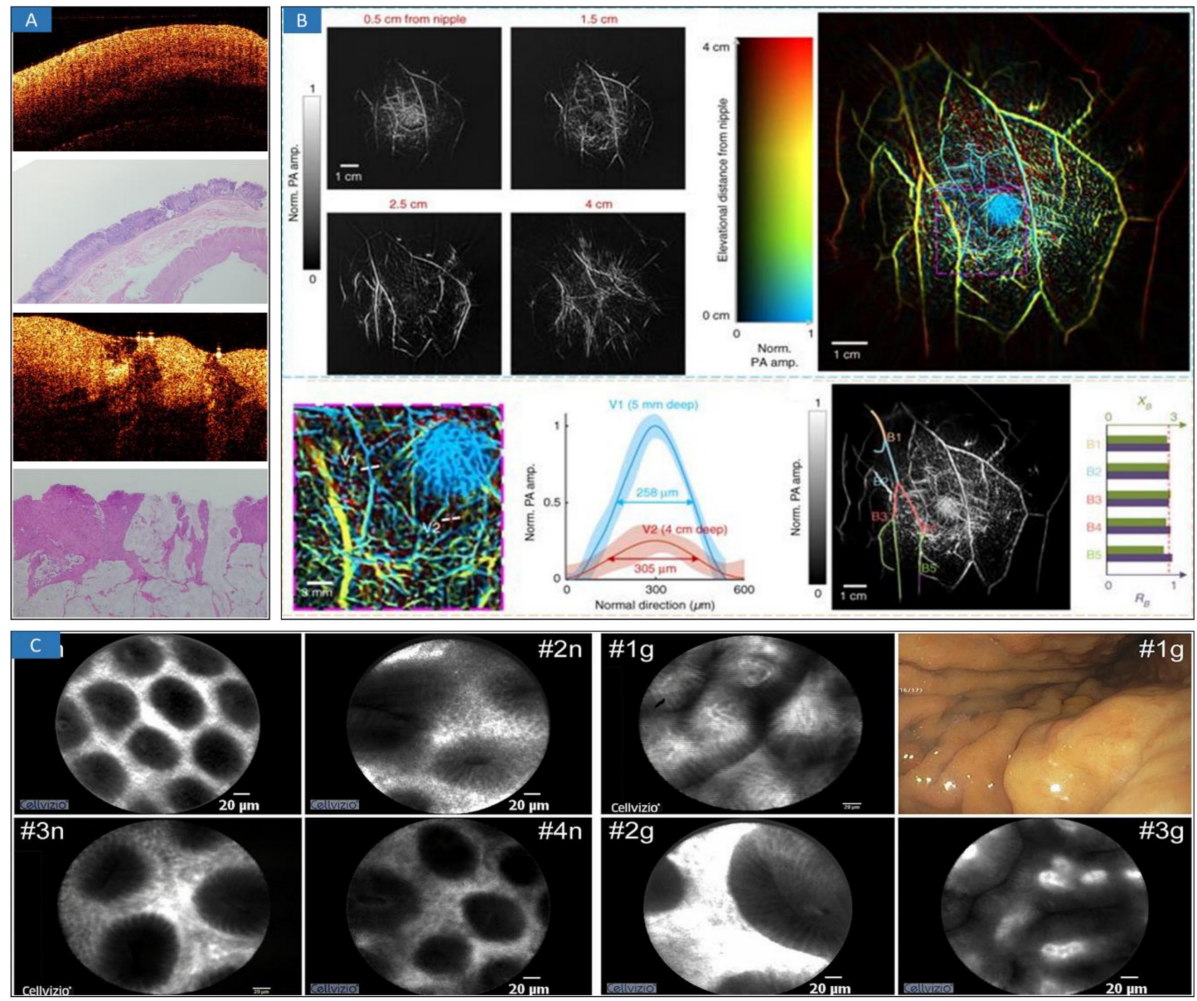
Optical minimally-invasive diagnosis. **A** OCT images and histology of normal tissue and cancerous tissue. Adapted with permission from 52, copyright 2019 IOP Publishing. **B** SBH-PACT of healthy breasts. Vasculature in the right breast of a 27-year-old healthy female volunteer. Adapted with permission from 71, copyright 2018 Springer Nature. **C** CLE images of a normal gastric mucosa (“n”) and mild gastritis (“g”). Adapted with permission from 76, copyright 2021 Springer Nature.

OCT has been widely used in the clinical diagnosis of disease theranostics 38-46, which usually focuses on ophthalmology, skin, and cardiovascular disease diagnosis 47. Due to the good performance of high-speed and high-resolution imaging, OCT-mediated intraoperative imaging has great potential in clinical cancer treatment 48. In terms of brain tumor diagnosis, OCT can provide real-time imaging and intelligent identification of cancers 27,29. For the diagnosis of breast cancers, the possibility using deep neural networks (DNN) of margin assessment has been investigated 49, demonstrating performance significantly higher than other reported results and close to the level of a human expert. Recently, optical coherence elastography (OCE) enabled the *in vivo* detection of residual breast cancer in the surgical cavity during breast-conserving surgery (BCS) 50. In a first-in-human study, twenty-one BCS patients were scanned *in vivo* with an OCE probe by five surgeons. The results indicate that *in vivo* quantitative microelastology can identify residual cancer by directly imaging the surgical cavity. Furthermore, optical characterization 51,52 (**Figure 2A**) is a useful parameter in evaluating tissue features for identifying cancerous and normal tissues based on optical transmission model. *En-face* polarization-sensitive OCT (PS-OCT) can be applied to effectively characterize protruding, flat, and depressive type esophageal squamous cell carcinoma in both animal and human specimens 53. In addition, the tumor margin could be drawn and determined on a broad *en face* view. The determined tumor margin could be in the proximity of 2 mm to the actual margin, which was proven directly using histology. Intelligent classification and segmentation methods, including deep learning 28,50,54-59 and other methods 60,61, could promote the detection and diagnosis of cancerous tissues.

Endoscopic OCT (EOCT) imaging 62 could promote the effective diagnosis of intraluminal tumors, such as gastric tumors and tracheal tumors. To emulate simultaneous clinical procedures and characterize healthy and diseased tissues, a 23 cm by 23 cm optical phantom was developed 66 to mimic the thickness and near-infrared optical properties of each anatomical layer of a human colon. The surface topography of colorectal polyps and visual appearance are acquired by endoscopic OCT compatible with white light endoscopy. Microelectromechanical systems- (MEMS-) based OCT probes have also been investigated 62,67 and can realize circumferential scanning of the intravital structure. The MEMS mirrors have a 0.5 mm × 0.5 mm mirror plate and a chip size of 1.5 mm × 1.3 mm. Full circumferential scans have been demonstrated based on different numbers of MEMS mirrors, typically two or six MEMS scanning units. Furthermore, a miniaturized OCT catheter was manufactured (outer diameter of 3.8 mm, lateral resolution of ~7 µm, and axial resolution of ~6 μm) 68. A passive, single-fiber probe in the gastrointestinal (GI) tracts of unsedated human patients 69 has been demonstrated and can be used to guide device placement. This probe conducts device-tissue physical contact sensing and obtains two-dimensional (2D) OCT images via M- to B-mode conversion. A customized ResNet is utilized to classify these OCT catheter colorectal images for automatic image processing and real-time diagnosis. An area under the receiver operating characteristic (ROC) curve (AUC) of 0.975 was achieved in distinguishing between normal and cancerous colorectal tissue images.

The real-time imaging feature of OCT enables doctors to observe and intervene in a timely manner, especially during ophthalmic surgeries and biological tissue sampling processes. OCT can be combined with other imaging techniques such as ultrasound or fluorescence imaging to provide more comprehensive information and improve diagnostic accuracy. Compared with radiation imaging such as X-ray imaging, OCT does not produce radiation, making it safer. Furthermore, the imaging depth of OCT is limited and suitable for observation of shallow tissues. Endoscopic OCT can display the fine structures inside organs, which is helpful for the diagnosis of early diseases within organs, such as intestinal mucosal abnormalities and esophageal lesions. However, although EOCT is relatively mild, it still requires the insertion of an endoscope, which may cause complications such as discomfort, bleeding, or infection. In addition, the use of EOCT requires experienced doctors to ensure correct navigation and image acquisition. Currently, more and more research on image navigation technology in endoscopic surgery is expected to break away from this limitation.

PAI was developed to provide effective and functional information regarding breast tissue 70,71 and brain tissue 72,73. A single-breath-hold photoacoustic computed tomography (SBH-PACT) system 70 (**Figure 2B**) was investigated and revealed detailed angiographic structures in human breasts. SBH-PACT features a deep penetration depth (4 cm *in vivo*) with high spatial and temporal resolutions (255 μm in-plane resolution and a 10 Hz 2D frame rate). Recently, real-time three-dimensional (3D) contour scanning of tissue via ultraviolet photoacoustic microscopy in the reflection mode was used to intraoperatively evaluate undecalcified and decalcified thick bone specimens 73. This method may allow for the rapid diagnosis of bone-tissue pathologies and aid the intraoperative determination of tumor margins. Furthermore, miniature microfibers with a large evanescent field encapsulated in polydimethylsiloxane (PDMS) 74,75 provide an excellent platform for the interaction between optical signals and ultrasound waves, exhibiting a high sensitivity of 3.5 mV/kPa, which is approximately 10 times higher than that of single-mode fiber sensors. The highly sensitive microfiber ultrasound sensor provides a competitive alternative for various applications, such as industrial nondestructive testing, biomedical ultrasound, and photoacoustic imaging.

Confocal microscopy has been used in the diagnosis of gastric cancers 76 (**Figure 2C**), such as confocal laser endoscopy (CLE). This high-resolution, noninvasive imaging technology provides the ability to microscopically evaluate cellular and subcellular features in tissue *in vivo* by optical sectioning 77. Because many cancers originate in epithelial tissues accessible by endoscopes, confocal endomicroscopy 78,79 has been explored to detect regions of possible neoplasia at an earlier stage by imaging morphological features *in vivo* that are significant in histopathologic evaluation and screening.

Other types of optical diagnosis are emerging over times with the innovation of optical technology, such as light-sheet microscopy 80, Mueller matrix imaging 81, and near-infrared fluorescence (NIRF) 82. Multimodal optical images are a hot topic in current research 83,84. A tri-modality imaging system 85,86 that integrates US, OCT, and NIRF for pancreaticobiliary duct imaging has been proposed. Similarly, an integrated PAT, OCT, and OCTA system has been developed to extract the human cutaneous microvasculature. This multimodal system has been demonstrated as a valuable tool for comprehensive noninvasive human skin vasculature and morphology imaging *in vivo*. Similarly, a dual modality imaging system that integrates PAT and OCT has also been developed and is like OCT and autofluorescence imaging 87. These systems have great clinical potential in mechanistic studies of real-time light-mediated treatment, early diagnosis, and prognosis evaluation of malignant pancreaticobiliary carcinomas. Endoscopic optical diagnosis could be a potential effective solution for noninvasive cancer diagnosis in MIT, such as endoscopic multimodal imaging systems 88. Hence, optical diagnosis could be more widely used in the clinical realm in the future.

Recently, the rise of deep learning has greatly increased the applicability of optical imaging diagnosis and improved its diagnostic accuracy. Deep learning models can be used to automatically segment different tissue layers in optical images and identify disease markers; optical images can also be classified to help doctors diagnose diseases. It can also help doctors monitor the progress of patients' diseases. Finally, deep learning technology can improve the quality of optical images, reduce noise, and improve the interpretability of images. However, the labeling cost of optical images is high, and the dataset is relatively limited. The model performs well on specific datasets, but its generalization to other datasets or clinical practice may be unstable. In addition, the successful integration of deep learning technology into clinical diagnosis requires appropriate norms.

### 2.2 Optical therapy

Optical therapy includes photothermal, photomechanical, and photochemistry treatments. There are three important methods as follows: laser ablation, PDT, and PTT. Currently, laser ablation and PDT are mainstream clinical applications. Laser ablation is widely used in current clinical applications 89. Through photothermal refection and vaporization, biological tissues can be effectively removed and ablated. Other optical therapies, such as laser cutting and laser osteotomy 90, are promising for future clinical applications.

**Figure 3:**
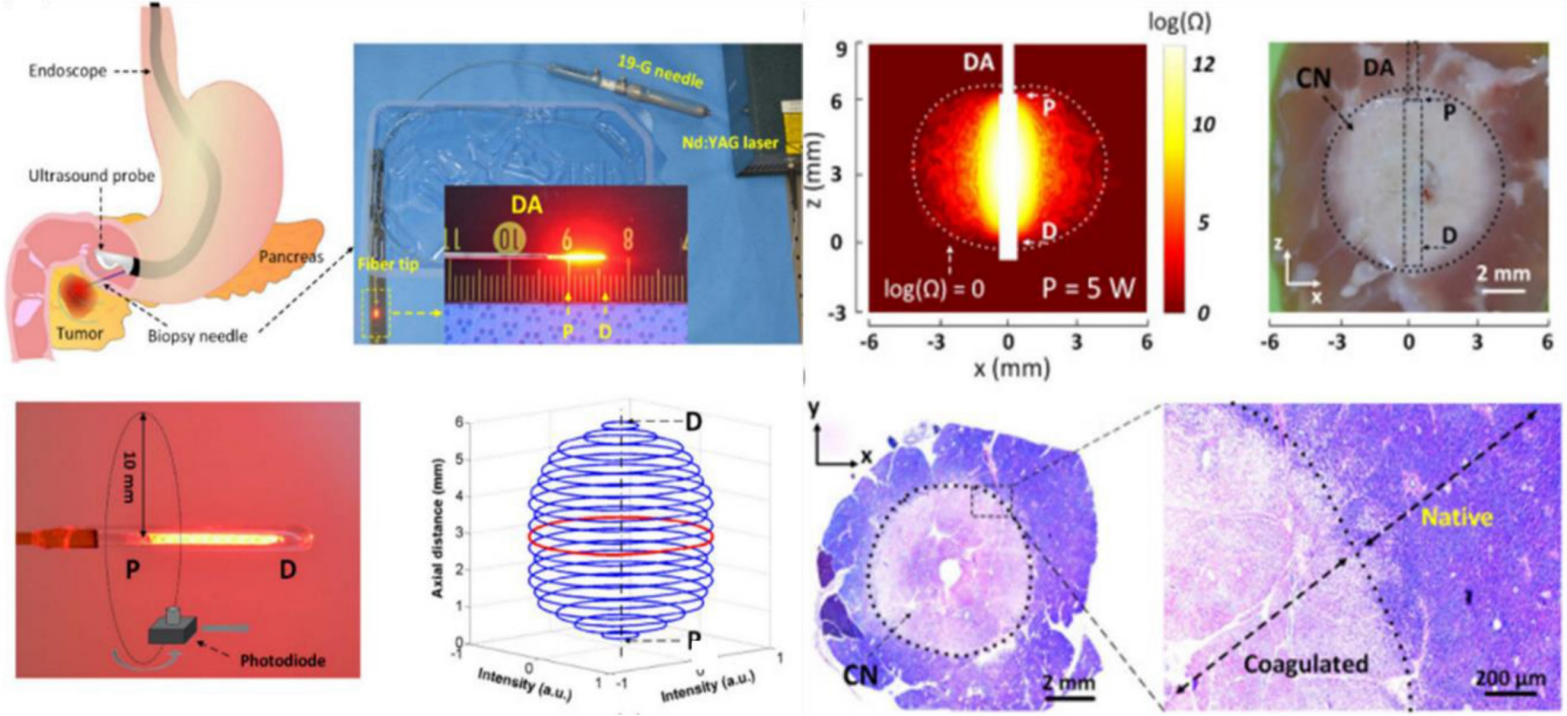
Laser ablation for cancer treatment. Circumferential interstitial laser ablation (CILA) of pancreatic tissue; illustration of endoscopic ultrasound (EUS)-guided CILA of pancreatic adenocarcinoma with a diffusing applicator through a small biopsy needle (19-G), uniform HeNe light distribution along a diffusing applicator, and 3D normalized spatial emission profile measured by photodiode. Adapted with permission from 101, copyright 2021 Optica Publishing Group.

Laser ablation is a solution involving the surgical removal or direct radiation of a tumor using laser technology to effectively eliminate tumorous tissue. By applying direct radiation with a high-power focused laser, tumorous tissue can be rapidly changed into vaporization and carbonization, thus eliminating the tumorous tissues. In the clinic, laser therapy is mainly applied for the treatment of tumors in the skin 91 and head and neck 92 and in neurology departments 93, but lasers are also suitable for the treatment of liver 94, lung 95, stomach 96, kidney 97, intestine 98 and other internal tumors 99. For the treatment of pancreatic adenocarcinoma, laser ablation under the guidance of endoscopic ultrasonography (EUS) 100 has been introduced, and the results demonstrate that, complete tumor ablation (CTA) was achieved in 94% of the patients in the US-laser ablation group and 100% of the patients in the EUS-laser ablation group (p=0.261). To demonstrate the feasibility of laser ablation, an EUS-guided PC ablation technique with enhanced safety was developed 101 (**Figure 3**). *Ex vivo* tests showed that both the ablation thickness and volume increased linearly with the applied power without carbonization and fiber degradation.

The treatment mechanism of laser ablation is very important for exploring the laser-biological tissue effect and thermal propagation during laser treatment. A laser hot needle has been proposed for tissue tract ablation 102,103 that is powered by a 4500-nm-diode laser incorporated with a closed loop control system that comprises a uniform fiber Bragg grating (FBG) temperature sensor and a computer. The laser power based on the real-time feedback input from the FBG temperature sensor 104 is regulated by a proportional-integral-derivative (PID) control system to control the needle temperature. To improve the strength and quality of laser tissue welding, the influence of temperature on the welding strength of biological tissue during the welding process was explored to establish a model of the relationship between welding temperature and tensile strength 105. In addition, OCT and infrared thermal imagers are applied to monitor the morphology and temperature during laser ablation 106. The results demonstrated that there was a good linear relationship between the radiation duration and temperature variation. Furthermore, the numerical simulations 107,108 match the experimental results in terms of temperature development and thermal damage. Measuring the interaction effect between light (or lasers) and biological tissue is very difficult during the laser ablation of diseased tissues. Numerical simulation and blackbody measurement may be useful methods for obtaining the relevant parameters in the process of laser ablation interaction 109-111. Hence, real-time monitoring and numerical simulation would be important methods for studying the mechanism and precision treatment of laser ablation for cancer treatments. Furthermore, real-time temperature monitoring is also important during LA. Infrared monitoring is a useful method to sensing the temperature of biological tissues' surface. MRI and FBG are treated as depth-related temperature monitoring methods. With automatic temperature regulation algorithms and estimation of thermal parameters, these methods would be a good solution of laser treatment for cancer therapy.

**Figure 4:**
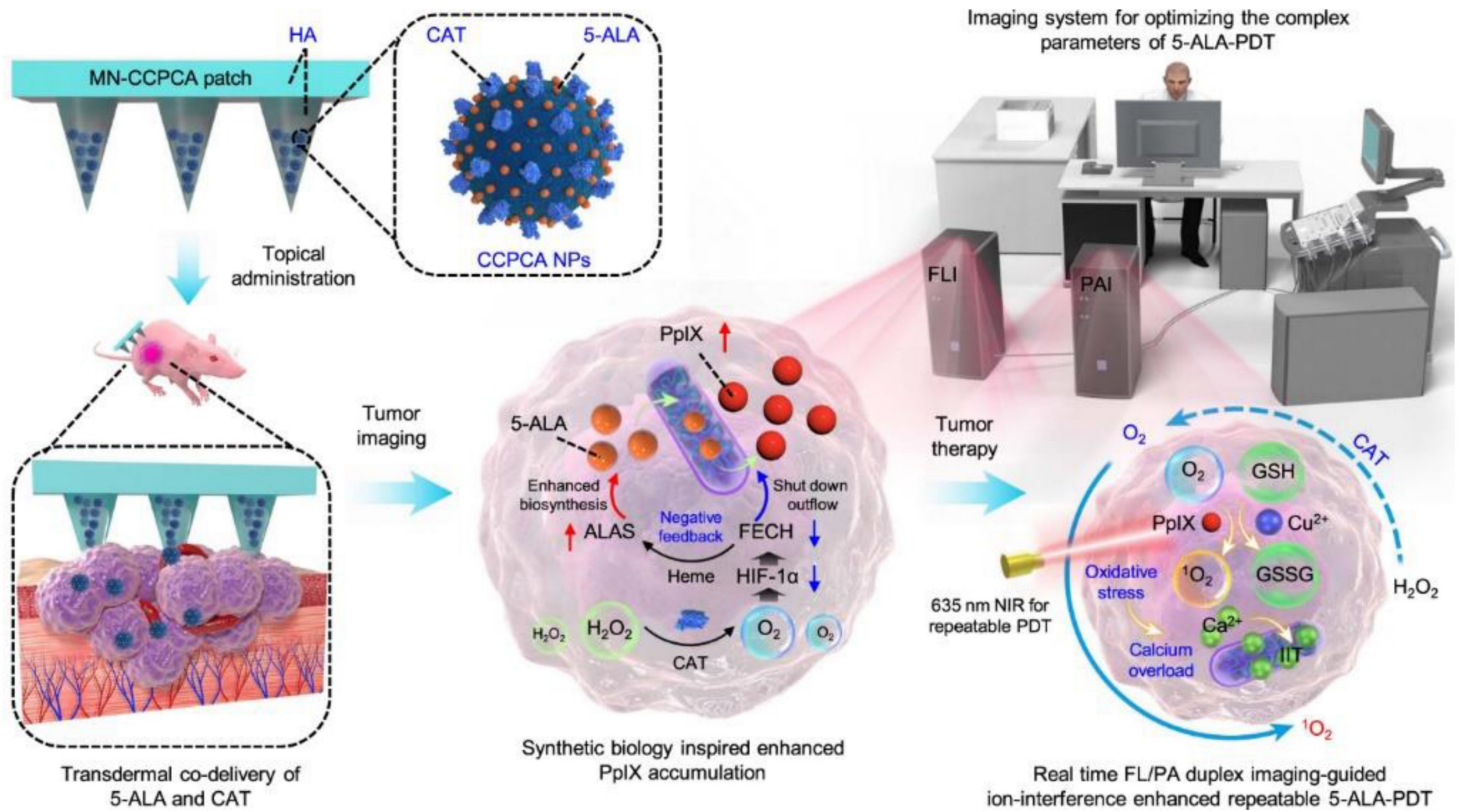
PDT and PPT for the treatment of cancers. *In vivo* real-time companion theranostics by a MN-CCPCA patch. The MN-CCPCA patch platform offers a strategy to monitor *in vivo* real-time PpIX and sO_2_ levels and optimize treatment parameters through duplex FL and PA imaging, ultimately improving the therapeutic efficacy and biosafety of 5-ALA-PDT. Adapted with permission from 116, copyright 2022 Springer Nature.

PDT started approximately in 1960 by Lipson 112, who adopted a photosensitizer and a relevant red-colored laser to destroy tumor cells in terms of single oxygen. PDT is being tested in the clinic for use in oncology 113 to treat head and neck, brain, lung, pancreas, intraperitoneal cavity, breast, prostate, and skin cancers. How does PDT work, and how can it be used to treat cancer 114? This seems to be a difficult question and be still a substantial challenge in clinical cancer treatment due to the low-level targeting and specificity of photosensitizers. Recently, a synthetic biology approach 115 was proposed to construct a transdermal theranostic microneedle patch integrated with 5-ALA and catalase coloaded tumor acidity-responsive copper-doped calcium phosphate nanoparticles by maximizing the enrichment of intratumoral protoporphyrin IX. A new form of PDT has been demonstrated 116 (**Figure 4**) in which nonlinear optical interactions of laser radiation with a biological medium *in situ* produce light that falls within the absorption band of the photosensitizer. Photosensitizers combined with functional microrobots 117 are being explored in medical fields. PPT is a treatment method that injects materials with high photothermal conversion efficiency into the human body 118, uses targeted recognition technology to gather near tumorous tissue, and converts light energy into heat energy under the radiation of external light sources (generally near-infrared light) to kill cancer cells. PTT usually results in localized thermal damage to normal tissues 119. Exogenous photothermal contrast agents 120 are not required for PTT but can enhance the efficiency and efficacy of treatment. To further improve the survival rate of cancer patients and reduce possible side effects in other parts of the body, it is still necessary to explore PTAs (**Figure 3**) with high selectivity and precise treatment 121,122.

Currently, some flexible and controllable endoscopes can be used to assist laser ablation 123, PDT, and PDD 124 in achieving efficient tumor removal. The integration of lasers with robotic control represents the next frontier in laser microsurgery. The device is 6 millimeters in diameter and 16 millimeters in length and can focus and steer a fiber-delivered laser beam at high speed (1.2-kilohertz bandwidth) over a large range (over ±10 degrees in both axes) with excellent static repeatability (200 micrometers) 123. A new flexible laser endoscope that integrates PDD and PDT and provides a controllable laser radiation field for the surgeon. Experimental results proved that the resolution of both diagnosis and treatment images was five times higher than that of standard laparoscopy 124. With the assistant of flexible device, optical therapy would will play a bigger role for the treatment of cancers.

## 3. Optical imaging-guided minimally-invasive therapy

### 3.1 Optical image-guided MIS

Optical image-guided MIS is a promising technique for adequately detecting tumor margins by tumor-specific targeting and effectiveness, potentially resulting in the complete resection of tumor tissue with improved survival and quality of life 125. Optical molecular imaging 126 for tumor detection severely depends on the imaging probes, which have been developed to target these biomarkers to improve the tumor contrast over the background tissue 127. Robot-assisted tissue surgery provides supervised autonomous operation 128. Due to limitation in the probe's safety and effectiveness, such surgery has a small number of clinical applications. Furthermore, the identification of the photons from the fluorescent contrast agent is complicated by autofluorescence, optical tissue properties, and accurate fluorescent targeting agents and imaging systems. Recently, some high-contrast fluorescent contrasts have been proposed. Aggregation-induced emission luminogens (AIEs) represent a promising probe for enhancing the imaging contrast of soft tissue tumors 129. However, these contrasts have only been explored in animal experiments and are currently difficult to apply in clinical practice. Multimodal optical image-guided minimally-invasive therapy 130,131 is an important development direction in the future. By the fusion of multimodal optical images, more abundant biological-tissue information and a greater surgical field of view (FOV) can be obtained effectively.

Computer- and robot-assisted navigation are important development directions for optical image-guided MIS 132,133. A supervised autonomous three-dimensional (3D) path planning, filtering, and control strategy for smart tissue autonomous robot (STAR) was developed to enable precise and consistent incisions in complex 3D soft tissues 134. The proposed strategy reduces the surface incision error and depth incision error by 40.03% and 51.5%, respectively, compared to a teleoperation strategy via the Da Vinci system. A concept of automated augmented reality (AR) registration, while the organs undergo deformation during surgical manipulation, is presented based on finite element modeling (FEM) coupled with optical imaging of fluorescent surface fiducials 135. The mean measured distance between the estimated tumor by biomechanical propagation and the scanned tumor (ground truth) was 0.84 ± 0.42 mm. Indocyanine green (ICG) for fluorescence image-guided minimally-invasive treatment of cholangiocarcinoma and hepatocarcinoma has been demonstrated to increase safety 136-137. Preliminary clinical results demonstrated this technique of NIR cholecystocholangiography by intragallbladder ICG injection during laparoscopic cholecystectomy 136. It can be considered in difficult cases to increase the safety of laparoscopic cholecystectomy. The feasibility of fluorescence liver segmentation by superselective intrahepatic arterial injection of ICG has been evaluated 137. ICG plays an important role in MIS or MIT. Intrahepatic arterial ICG injection rapidly highlights hepatic target segment borders with a better signal-to-background ratio (SBR) than portal vein injection. Therefore, ICG is an effective tool to enhance the tumorous tissues, while, the tumor boundary is still unclear. This question is a challenge in clinical applications, more targeted contrast and automatic identification algorithms would be developed for enhancing the SBR and clear boundary of diseased tissues to future improved clinical applications.

### 3.2 OCT-guided laser ablation

**Figure 5:**
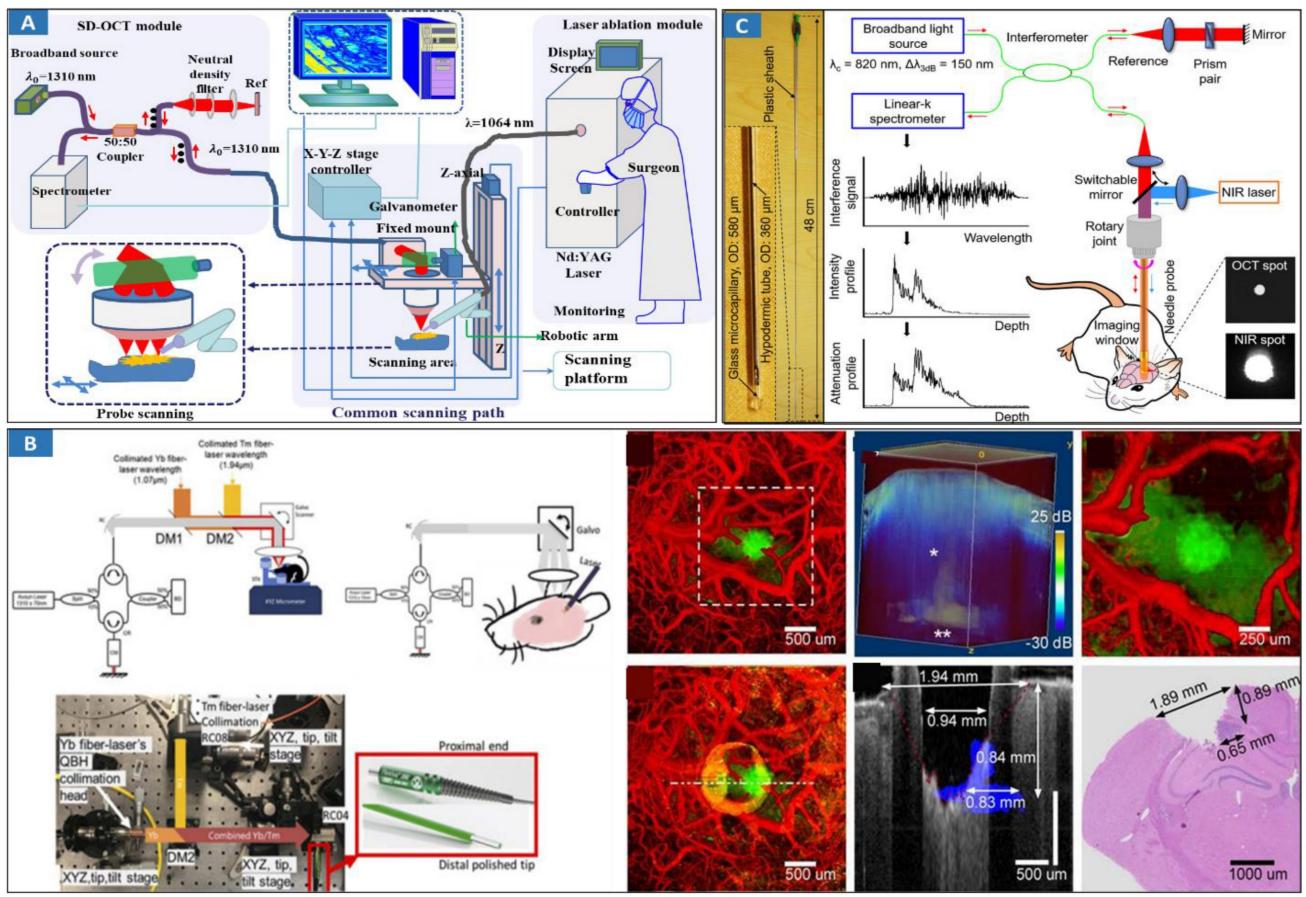
Optical MIT based on OCT-guided laser ablation for cancer treatment. **A** Principal schematic of the integrated SD-OCT and laser ablation system. The system includes an SD-OCT unit, laser ablation module, and endoscopic scanning platform module. Adapted with permission from 140, copyright 2018 Elsevier. **B** Benchtop OCT-guided laser surgery system with coaligned Tm/Er beams. Tm/Er and OCT beams are fiber delivered via reflective collimators (RC) and combined with di-chroic mirrors (DM). Histology of a 5 μm thick transverse section located 550 μm from the cortical surface, and B-scan of tumor regions (blue). Adapted with permission from 143, copyright 2018 Ivyspring International Publisher. **C** Theranostic microneedle system with ultrahigh-resolution OCT imaging and laser ablation. Photograph of the microneedle with a hypodermic tube of a 360-m OD and a 0.48-m rigid length. Adapted with permission from 150, copyright 2020 AAAS publications.

In 1999, OCT-guided laser ablation for minimally-invasive treatment was proposed for the first time 139. Real-time OCT imaging is used to guide placement and observe the dynamics of surgical laser ablation in a variety of tissue types. OCT-guided laser ablation of brain tumors was proposed in our previous research 140 (**Figure 5A**). OCT, as a fast and high-resolution imaging method, is used to monitor and sense laser surgery and ablation 141. Usually, OCT imaging is only used as a mean of monitoring but not as an effective method of intelligent intraoperative diagnosis and guided real-time treatment. Recently, Nitesh Katta et al proposed the integration of OCT and laser ablation for the minimally-invasive treatment of cancers, and animal experiments were conducted in which phantom blood vessels 142 and animal brain tumors 143 were treated. The combination of high-resolution, fast OCT coaligned with a nanosecond pulsed thulium (Tm) laser 142 offers advantages over conventional surgical laser systems. A tissue removal rate of 5.5 mm^3^/sec was achieved experimentally in comparison to the model prediction of approximately 6 mm^3^/sec. The theranostic system 140,143 (**Figure 5B**) combines OCT guidance with surgical lasers for high-precision tumor ablation (Er: YAG) and microcirculation coagulation (thulium (Tm) fiber laser). Our previous research revealed that intelligent theranostics is an important development direction through intelligent robot assistance and precise surgical-path planning 144,145, and the surgical robot can reach the planned position with high precision, which is approximately 1.16 mm. Experiments show that the proposed system is capable of clearing lesions efficiently and precisely. Intelligent OCT-guided laser ablation provides feedback information reflecting the intraoperative situation in real time 146. But, this method is adjustable open surgery, and for minimally invasive surgery, it needs to be reconsidered.

A fiber-laser platform integrated with OCT imaging has also been developed for precision brain surgery 147. Two-fiber lasers were combined into a single biocompatible silica fiber to conduct brain surgery resection under the bench-top OCT system's imaging microscope. Thermal injury was measured to be less than 100 μm, while the removal rate was close to 5 mm^3^/s with an average Tm fiber-laser power of 15 Watts. Furthermore, OCT guided endoscopic laser surgery 148 is a hot topic for future clinical cancer treatments. Endoscopic theranostic catheter of the OCT and ablation has been proposed 149. The catheter consists of a fiber bundle including 41 multimode fibers with an outer diameter of 0.9 mm. A rigid theranostic probe was also developed by combining OCT and laser ablation 150 (**Figure 5C**). This theranostic needle's performance was demonstrated by *in vivo* ultrahigh-resolution (1.7 μm axial and 5.7 μm transverse), high-speed (20 frames per second) volumetric imaging of mouse brain microstructures and optical attenuation coefficients. Its translational potential was further demonstrated by *in vivo* cancer visualization and efficient tissue ablation in a deep mouse brain. This research implemented the hard theranostic endoscope with the OCT guided laser ablation rather than flexible or switchable-hardness endoscope as mentioned earlier, so it has some invasion during cancer treatment. As the development of material, mechanical, and information sciences, flexible endoscope with theranostic function using light-mediated diagnosis and therapy can be developed to provide minimally invasive invention and treatment by choosing approximate light energy. For example, switchable rigid-flexible robot endoscope would be integrated with fibers and MEMS mirrors to reflect the light of OCT-based diagnostic and ablation-based therapeutic to tissues using the common optical path method.

### 3.3 Fluorescence imaging-guided laser ablation

**Figure 6:**
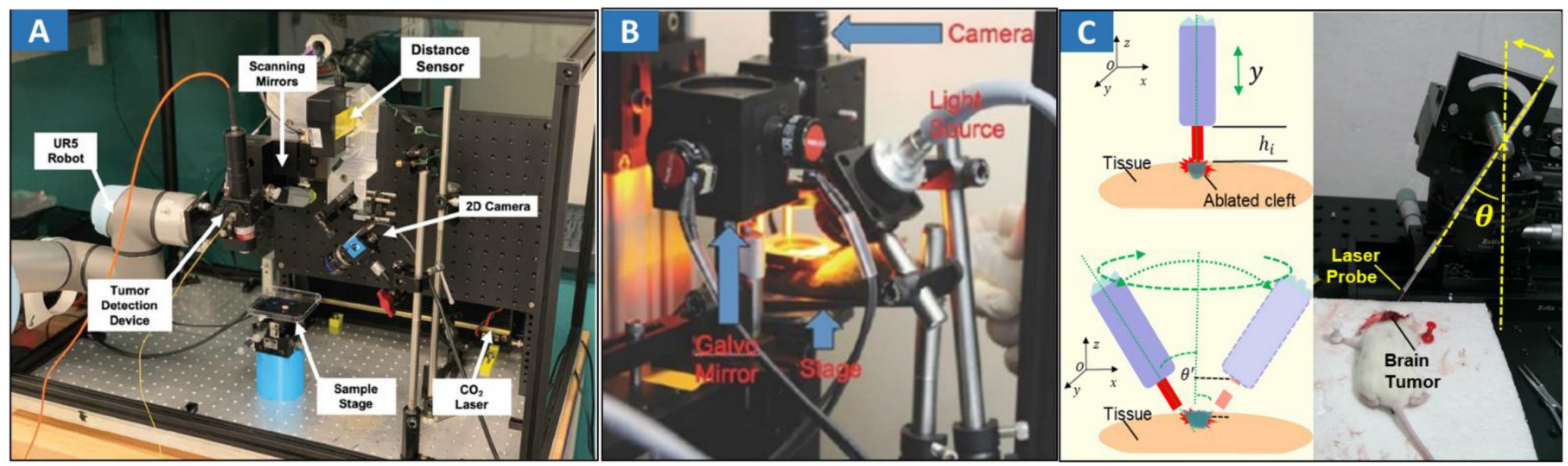
Optical MIT based on optical image-guided laser ablation for cancer treatment. **A** Experimental setup showing the TumorID positioned with the FOV of the TumorCNC by the UR5 robot. Adapted with permission from 151, copyright 2021 IEEE. **B** Paired imaging/laser ablation prototype and system characterization. The optical layout of the integrated image detection and laser ablation system is shown. A 1064 nm laser source is directed through a diffuser toward a series of motorized galvo-mirrors. A fiber bundle is connected to a xenon light source and attached to the system to illuminate the specimen. Adapted with permission from 152, copyright 2016 Ivyspring International Publisher. **C** Different heights between the fiber tip and tissue surface within the perpendicular incidence and different incidence angles of the fiber probe. Adapted with permission from 162, copyright 2018 Ivyspring International Publisher.

Fluorescence imaging-guided laser ablation provides a highly efficient treatment for cancers. 5-ALA fluorescence imaging has been incorporated into a robotic laser ablation neurosurgery system 32,33. The accuracy of the fluorescent measurement of the tumor was improved using a high-precision spectral analysis. Fluorescence assists in the detection of malignant brain tumors intraoperatively and improves their removal rate. A contactless tumor removal system that utilizes endogenous fluorescence feedback to inform the laser ablation system to execute the autonomous removal of phantom tumor tissue was created 151 (**Figure 6A**). This completely noncontact surgical system can resect the tumor boundary of a tissue phantom with an average root mean square error (RMSE) of approximately 1.55 mm and an average max error of approximately 2.15 mm. A fluorescence-guided laser ablation system for the removal of residual cancer has been proposed 152 (**Figure 6B**). This pulsed Nd:YAG laser ablation system, when used in conjunction with a molecular imaging system, can identify and ablate cancer *in vivo*. Laser ablation was guided by fluorescence imaging to target tumor tissues while avoiding normal structures.

CARS imaging has been combined with fs-laser ablation as a new approach for image-guided precision surgery 153 (**Figure 6C**). CARS-guided fs-ablation has been applied to ablate brain, liver, skin, muscular and vascular tissues with μm-level precision using sub 100 fs pulses at the μJ level. Superior imaging performance, contrast and detection of tissue margins is demonstrated by coherent Raman microscopy in comparison to laser reflectance imaging. A novel robotic device for intraoperative large-area endomicroscopy imaging and image-guided ablation has been proposed 154. The device also includes a laser ablation fiber to precisely ablate target tissue under the image guidance without the need for additional assistance. As mentioned earlier, tumor boundary could be not clear so that laser ablation cannot effectively treat the tumor. Enlarged ablation area is needed to totally eliminate tumorous tissues. Hence, classical assistant device should be developed.

### 3.4 Multimodal imaging guidance of laser ablation

Multimodal imaging can provide great information to guide laser ablation in the treatment of cancers. An integration of white light bronchoscopy (WLB), endobronchial ultrasound (EBUS), and OCT might identify *in vivo* airway wall changes before and those resulting from Nd:YAG laser ablation and dilation of tracheal stenosis 155. High-resolution multimodal PAT and OCT were developed to improve the efficiency of visualizing newly developed retinal neovascularization (RNV) and monitor the dynamic changes in retinal vein occlusion (RVO) 156. A novel photoacoustic (PA)-guided laser ablation theranostic device 157 was developed based on a traditional phased-array endoscope. The proposed technology exhibits the effective capabilities of lesion formation, tissue distinguishing, and temperature monitoring. An integrated photoacoustic imaging and high intensity focused ultrasound (HIFU) system 158 using a 5-ns tunable OPO laser system and a 5 MHz HIFU transducer was used to perform a photoacoustic analysis to identify the optical contrast and perform combined laser and ultrasound ablation 159. US-guided laser ablation systems 100 have been developed for pancreatic tissue. US is a useful theranostic tool, which also can focus on US imaging guided HIFU treatment, and provide real-time imaging and diagnostic information, however, US suffers the limitation of resolution and contrast, is difficult to image the approximate cellular-size tissues.

MRI-guided laser ablation is an exciting new minimally-invasive technology for the treatment of cancers. MRI-guided laser interstitial thermal therapy (MRgLITT) is a procedure for destroying tissue using heat effects. This approach can be used in the treatment of epilepsy 160 and hepatocellular carcinoma 161. The integration of MRI and bioluminescence imaging (BLI) can be used to guide laser ablation and provide quantitative information reflecting the laser ablation effect of carcinoma. Recently, we demonstrated that BLI and two-photon microscopy (TPM)-guided laser ablation of GBM decreased the tumor burden 162. The BLI quantitatively and qualitatively evaluated treatment using laser ablation with the appropriate laser parameters and laser-tissue parameters. The accuracy of the laser ablation reached a submillimeter level, and the resection ratio reached more than 99% under the guidance of BLI. In future, it is possible to be combined with some intelligent or smart robotic technologies toward high-precision treatment for tumors ablation. Furthermore, multimodal imaging can provide plentiful and abundant information to guide laser ablation. Such information includes cross-sizing structural, and functional features for representing the tissues and can be used to guide the precision treatment of diseased tissues.

## 4. Optical image-guided PPT and PDT

PPT/PDT is a useful and efficient solution for the treatment of soft tissue carcinomas. Combined with OCT/OCTA and other optical imaging modalities, the treatment effect and response of PDT/PTT could be clearly presented through the visualization of multimodal images.

### 4.1 OCT-guided PDT/PTT

**Figure 7:**
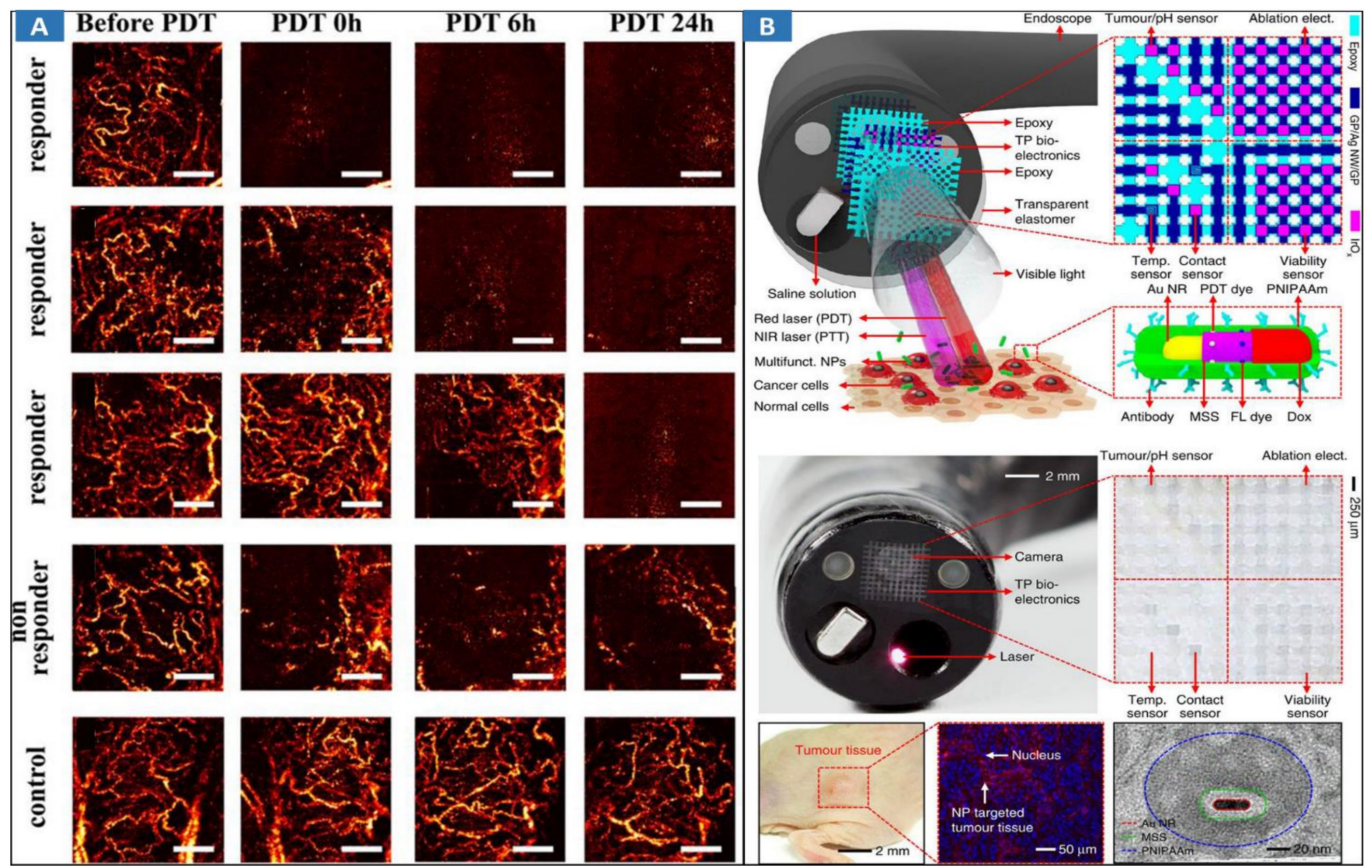
Optical MIT based on multimodal optical image-guided PPT/PDT for cancer treatment. **A** MML-OCA images of microvascular alteration dynamics prior to, immediately following, 6 hrs post, and a day after PDT (100 J/cm^2^, 100 mW/cm^2^). Adapted with permission from 169, copyright 2017 Springer Nature. **B** Multifunctional endoscope system. Schematic illustrations of the design strategy and mode of use of the multifunctional endoscope system based on transparent bioelectronic devices and theranostic nanoparticles. Images of the system corresponding to the illustrations. Adapted with permission from 8, copyright 2015 Springer Nature.

Due to the high resolution and rapid imaging speed, monitoring changes with OCT in blood flow dynamics and vessel structure following pharmacological intervention and PDT have been demonstrated 163-166. The technical feasibility of OCT has been demonstrated to map real tumor margins and monitor skin changes that occur post-PDT 167. The technical feasibility of catheter-based intraluminal Doppler OCT (D-OCT) 168 for monitoring the microvascular response during endoluminal PDT was assessed in an animal model of Barrett's esophagus (BE). Distinct microstructural differences between normal squamous esophagus, BE, and the transition zone were clearly observed on DOCT images. Similar submucosal microcirculatory effects (47%-73% vascular shutdown) were observed during PDT of the normal esophagus and surgically induced BE. The controls displayed no significant microvascular changes. PDT monitoring with optical coherence angiography (OCTA) 169 has been proposed (**Figure 7A**), and M-mode-like OCTA (MML-OCTA) was able to sensitively detect PDT-induced microvascular alterations. OCTA for pretreatment assessment and treatment monitoring following PDT in patients has also been researched 170. Pretreatment OCTA enabled differentiation between prevalent subtypes of BCC (nodular and superficial) and nodular-with-necrotic-core BCC subtypes with a diagnostic accuracy of 78%, which can facilitate more accurate biopsy, reduce the sampling error, and improve therapy regimen selection. Posttreatment OCTA images at 24 hours were 98% predictive of the eventual outcome. To assess the early tumor reaction and predict its long-term response, the combination of OCTA and compressional OCE enables complementary functional/microstructural tumor characterization 171. Despite the different mechanisms of antiangiogenic action of antiangiogenic chemotherapy (ChT) and PDT, in both cases, OCA demonstrated high sensitivity to blood perfusion cessation. The novel method of OCE-based morphological segmentation revealed very similar histological structure alterations. To guide and assess PDT treatment, a handheld OCT probe was designed for real-time imaging of the PWS patient 172. The system also has a spatial resolution of 8 μm (lateral) × 7 μm (axial), an imaging rate of four frames per second, and a 102 dB sensitivity. Functional monitoring of PDT for cancer treatment could provide useful information during and after PDT. OCT can use the characteristics of the tissues' Doppler effect, elastic properties, and angiography to detect the structural and vascular features of biological tissues treated by PDT method, then, monitor the changes during PDT.

### 4.2 Multimodal optical image-guided PDT/PTT

Multimodal optical image-guided PDT/PTT for cancer treatments has been widely researched 119,173-176. ICG-guided photothermal ablation using a one-step method has been proposed for the preparation of holo-Tf-indocyanine green (holo-Tf-ICG) nanoassemblies for fluorescence and photoacoustic (PA) dual-modal imaging and PTT of glioma. Under near-infrared laser radiation, the holo-Tf-ICG nanoassemblies accumulated in tumor regions can efficiently convert laser energy into hyperthermia for tumor ablation. The novel theranostic nanoplatform holds great promise for precision diagnosis and the treatment of glioma. An endoscope with integrated transparent bioelectronics and theranostic nanoparticles has been developed for colon cancer treatment (**Figure** 7**B**) 8, and the nanoparticles are photoactivated within a highly localized space near tumors or benign growths. These advanced electronics and nanoparticles collectively enable optical fluorescence-based mapping, electrical impedance and pH sensing, contact/temperature monitoring, radiofrequency ablation and localized photo/chemotherapy as the basis of a closed-loop solution for colon cancer treatment. This work presents excellent MIT framework, which integrates the effective endoscopic imaging and therapy. However, it suffers from some demerits, such as the safety of theranostic nanoparticles, and the lack of endoscopic control strategies for MIT.

Photoacoustic imaging 177 and near-infrared light-triggered 178 theranostic platforms have great potential in applications, such as cancer-targeted fluorescent imaging and simultaneous ROS-activated chemo- and PDT in the near future. A multifunctional theranostic contrast agent 179 is presented for ultrasound/near infrared fluorescence imaging-based tumor diagnosis and ultrasound-triggered combined photothermal and gene therapy. The developed theranostic AuMB complexes could not only provide excellent US and NIRF imaging to detect tumors but also serve as an efficient US-triggered carrier for gene delivery and photothermal ablation of tumors in xenografted nude mice. Multimodal image-guided surgical and photodynamic interventions 180 have been proposed, demonstrating that multimodal porphyrin lipoprotein-mimicking nanoparticles (PLPs) intrinsically capable of PET, fluorescence imaging, and PDT show great potential to enhance the accuracy of HNC staging and potentially head and neck cancer (HNC) management. PDT is an effective adjuvant therapy for image-guided surgery in prostate cancer 181, demonstrating that the prostate-specific membrane antigen (PSMA)-targeted PDT agent PSMA-1-Pc413 selectively highlights PSMA-expressing tumors, allowing IGS and more complete tumor resection compared with white light surgery. However, due to the safety and effectiveness of nanomaterials, nanomedicine 182 is difficult to apply in clinical diagnosis and treatment. Currently, photosensitizers approved clinically have important applications in tumor treatment. Multimodal optical imaging, such as OCT, confocal endoscopy, PAT, and narrowband spectral imaging (NBI) 183, combines photosensitizers to display the whole and wide FOV and present real-time dynamic information during PDT.

## 5. Outlooks and challenges

In this paper, we outlined emerging optical MIT platform based on optical image-guided optical treatments for MIS of cancers. Comprehensively, we summarized the research of MIS, laser ablation, PDT, and PTT in LMIT in detail and provided some objective evaluations in relevant positions. LMIT toward intelligent medical devices could provide a solution for clinical applications. Optical imaging has a high resolution, is fast and noninvasive, has other important imaging characteristics and has important clinical application value. Furthermore, label-free optical imaging is safe and effective in clinical practice. It can present the anatomical structure of biological tissue and other high-resolution images (µm level) 27,28,38-48 without a contrast agent (label-free) and can provide functional images of biological tissue. Optical imaging can provide the elastic coefficient (OCE) 184 of biological tissues, blood flow in blood vessels (OCTA, and DOCT) and other functional information, which has very important clinical significance in the recognition of soft tissue diseases. Inventors of OCT is recently awarded the 2023 Lasker-DeBakey Clinical Medical Research Award 185. This honor has encouraged more researchers to apply OCT to a wider range (including non-invasive, high-speed, and label-free imaging) of biomedical applications, and to develop some novel light-based medical devices to clinical cancer treatment. Optical therapy, such as laser ablation, has great advantages in minimally-invasive targeted therapy of tumor tissues due to its characteristics of minimal invasiveness, radiation free and good targeting. However, the photobiological effects in the process of PTT need to be further clarified, especially the biological effects, photothermal propagation model, photochemical effects, etc., which could provide theoretical support for the mechanism of optical treatment.

Optical theranostics 144,145,162 include digitalized precision diagnosis, treatment, and their integration with automatic and intelligent diagnosis and treatment of diseased tissues under or without surgical assistance, which can be guided by laser ablation for cancer treatment. In terms of imaging and intelligent diagnosis, due to the limitation of the optical imaging depth (several-millimeter levels), enhancing the imaging depth is an important direction of future development, including popular optical transparency technology, which enables optical imaging diagnosis and treatment technologies to extend several millimeters in the longitudinal direction 186. Intelligent algorithms for medical optical images can draw tumor boundaries and provide tumor grading information in the process of tumor imaging and diagnosis. With the continuous development of artificial intelligence and deep learning algorithms 50,58,59, fusion and registration based on optical images and other traditional medical images have already had extensive clinical application foundations in image-aided diagnosis, and could play a better and more extensive role in MIT for cancer treatment in the future.

Furthermore, multimodal optical images 187 provide effective and comprehensive intraoperative information regarding biological tissues, including cross-scale structural and functional information. OCT and photoacoustic imaging can complement each other in imaging depth and resolution. In the process of PDT and laser ablation, the presentation of structural and functional information provides an effective quantitative evaluation method for the clarity of intraoperative treatment. Cross-scale optical imaging could provide imaging effects on different scales for tissue precision diagnosis. Fluorescence imaging can present organ-size images; photoacoustic imaging can present submillimeter images; OCT can provide micrometer-level images of cancerous tissues; and microscopic optical imaging, such as confocal microscopy, can provide submicrometer-level images. The imaging depth and imaging resolution are contradictory. The integration of cross-scale imaging modalities can solve this problem 188.

In terms of light-mediated treatment, the mechanism of laser ablation based on NIR and visible light should be further explored. By combining numerical simulations 101,107,108,191, the thermal effect and propagation of laser radiation can be treated as an access to analyze the treatment effect on soft tissue carcinomas. The therapeutic mechanism could be established effectively to promote the further development of the theory of LMIT. In addition, the development of novel optical materials could lay a solid foundation for optical imaging and targeted optical therapy. These materials enhance the imaging effect, including the imaging contrast, and strengthen the foreground of cancerous tissue's optical images. Furthermore, these materials could enhance the targeted feature for accumulation in cancerous tissues to intensify the treatment. However, the biological safety of these novel materials is a major challenge for novel materials. Prior to clinical application, it is urgent to carry out effectiveness verification research to determine the effectiveness and biological safety of novel materials and further prove their biological safety in humans in clinical trials.

The application of laser ablation in tumor therapy has some significant advantages. Firstly, laser ablation is a non-invasive treatment method, which can avoid the incision and trauma of traditional surgery, and reduce the pain and recovery time of patients. Second, laser ablation has less damage to surrounding normal tissues and relatively protects the integrity of healthy tissues. It can accurately locate and target tumor tissue *in vivo*, enabling precise treatment. However, laser ablation also has some limitations and risks in tumor treatment. First, the method has certain limitations on the size, shape, and location of the tumor, and it is not suitable for tumors that are large or located at specific locations. Secondly, laser ablation may not achieve the effect of comprehensive tumor removal, and there is a risk of recurrence. In addition, laser ablation may also cause some complications, such as thermal injury, scarring, or paresthesias. In view of these limitations and risks, doctors need to comprehensively consider the specific situation of the patient and the characteristics of the tumor to determine the most suitable treatment plan. In some cases, laser ablation may be used in combination with other treatments, such as surgical resection, radiation therapy, or chemotherapy, to improve outcomes. Overall, laser ablation, as a promising technique of tumor treatment, has certain application prospects and advantages. However, treatment options should be individualized for each patient and require full discussion and evaluation with a physician to ensure optimal therapeutic efficacy and safety. Precise treatment 153,162 and intraoperative monitoring 157 using laser are currently the main research directions and have great potential for future development.

Different types of tissue have different penetration capabilities of laser. Tissues, such as skin, fat, and blood vessels, penetrate laser better than bone and joint tissue. Depth is also a key factor, as laser energy tapers off in tissue, and deep tissue penetration may be limited. Laser wavelength plays an important role in its ability to penetrate tissue 189. Different wavelengths of laser are absorbed to different degrees by different types of tissue. Infrared laser wavelengths, for example, are outside the visible range and penetrate certain tissues better 190. In addition, hemoglobin and melanin have high absorption capacity for laser at specific wavelengths, which may have an impact on certain therapeutic and diagnostic methods. Light scattering is an important phenomenon during the propagation of laser in tissue. Scattering causes spreading and attenuation of laser energy, limiting its penetration depth in tissue. The degree of light scattering is affected by the structural and optical properties of the tissue, such as the density and reflectivity of the tissue. Penetration in tissue is also limited by safety considerations. Higher energy lasers may cause excessive heating and risk of thermal damage to tissue. Therefore, in laser therapy, careful control of laser parameters is required to ensure a balance of safety and efficacy.

The ANSI Z136.1 standard plays an important role in laser safety management, providing guidance and requirements for the use and operation of laser equipment. However, users and operators should comprehensively consider relevant laws, regulations, and actual conditions when applying this standard to ensure the safe use of laser equipment and protect personnel and the environment from potential hazards of laser radiation. Furthermore, under the guidance of laser safety standard, medical laser and medical optical imaging can be safely researched and developed.

The automatic treatment of robotic-assisted surgery can remove the surgeon's hands, which promises enhanced efficacy and safety and improved access to optimized surgical techniques. *In vivo* supervised autonomous soft tissue surgery in an open surgical setting was enabled by a plenoptic three-dimensional and NIRF imaging system and an autonomous suturing algorithm 191. Autonomous surgical robots have the potential to improve the efficacy, consistency, functional outcome, and accessibility of surgical techniques 192. Through the automatic assistance of robot, optical imaging-guided microsurgery can be smoothly implemented after reinforcement learning 193. Currently, intelligent MIT equipment 194 can provide an effective solution in laparoscopic surgery for intestinal anastomosis. Intelligent theranostic equipment includes intelligent identification, analysis, diagnosis, intelligent minimally-invasive treatment, and theranostic methods. The most typical minimally-invasive theranostic methods are usually surgical navigation and IGS. The innovative research and development of surgical navigation and IGS technology effectively integrates minimally-invasive diagnosis and treatment schemes from the perspectives of engineering and provides an image-guided surgery algorithm and specific steps for the implementation of image-guided therapy at the design level of LMIT.

The design of hardware structures and optical path structures provides equipment support for the integration of diagnosis and treatment 195,196, especially in light source characteristics (wavelength, power, energy density, radiation time, etc.), optical path compactness, equipment integration, and system safety. In addition, in terms of treatment equipment assistance, the switchable rigid-flexible robot 197 can realize wide-field optical imaging of biological tissues and efficient intelligent treatment of tumors. Research investigating the theory and application of these aspects lays a solid foundation for the design and development of intelligent optical theranostic equipment in the future. However, in intelligent MIT, the current lack of surgical methods for specific sites and tumor resection and the selection of treatment plans are still urgent directions of research necessary for the personalized and precise treatment of soft tissue carcinomas.

The endoscopic theranostic platform 198 is a future development direction in LMIT and could promote the rapid development of MIS. Given the versatile role of light in nature, the many ways that light-based theranostic approaches can sense, monitor, and manipulate treatment processes are not surprising. Endoscopic OCT, photoacoustic CT, confocal microscopy, and hyperspectral imaging are some convenient tools reflecting *in vivo* intraoperative information 199, including structural and functional situations, for presenting the effective biological states to monitor the process of cancer treatment. Optical molecular imaging 200 could be rapidly used to acquire tumor-to-tumor molecular heterogeneity, both dynamically and quantitatively. Optical imaging can be integrated into endoscopic theranostic platforms for real-time sensing of therapeutic states and monitoring the progress of light-induced therapy. Furthermore, minimized interventional theranostic probes integrating optical diagnosis and optical therapy for precision cancer treatments could be an important direction for translational clinics, such as natural orifices or minimal incisions and intraluminal interventions. This solution has wide clinical potential for interventional treatment in the future.

With the research of various technologies in LMIT, it has been widely developed in clinical applications, providing patients with less pain, shorter recovery time, and lower tissue trauma. Different optical methods can be selected based on the patient's disease type and specific situation to achieve the best treatment effect. Each technology has its own indications and limitations, and there may be multiple diagnostic methods or collaborative diagnosis and treatment methods during the surgical process. Research on devices that integrate multiple diagnostic and treatment methods is still in the research stage, and further research is urgently needed to popularize them in clinical minimally invasive treatment.

## 6. Conclusions

Intelligent LMIT provides an optimized solution for the minimally-invasive treatment of tumorous tissues due to the excellent features of light-mediated theranostic strategies. This paper reviews the state-of-the-art light-mediated theranostics, which improve and update the treatment efficiency of tumorous tissues. Intelligent LMIT systems would been developed for use in clinical trials. Optical or light-mediated MIT equipment is an important developmental trend in future research and clinical practice. Such equipment provides the function of intraoperative imaging and diagnosis, surgical navigation, deep imaging-guided precision therapy, and intelligent minimally-invasive treatment. LMIT could provide great convenience, and improve and optimize the efficiency of cancer treatment.

## Figures and Tables

**Table 1:** Overview of topics and technical trends addressed in this review

Topic	Example terms/References	Key features	Trends/Future researches	Comprehensive comments
Optical diagnosis	Boppart SA *et al*, 40	Optical coherence tomography (OCT), and intraoperative imaging	Intraoperative imaging, and endoscopic optical diagnosis; Multimodal optical imaging, and combining with intelligent algorithm to realize the automatic identification	First-time intraoperative imaging and guidance
	Fan Y *et al*, 62-64	Endoscopic OCT		Endoscopic imaging for diagnosis
	Lin L *et al*, 71	Photoacoustic tomography (PAT)		First-time whole-body PAT
	Jabbour M *et al*, 76	Confocal microscopy		Classical pCLE in gastric cancer
	Patel KB *et al*, 80	Other types of optical diagnosis		*In vivo* high-resolution pathological examination
Optical therapy	Jermakowicz WJ *et al*,^89^	Laser ablation	Exploring the treatment mechanism of optical treatment, and need to be guided by medical image and robot	Simulation and prediction of laser ablation for tumor treatment
	He G *et al*, 116	Photodynamic therapy (PDT)		A transdermal theranostic microneedle patch
	Jivraj J *et al*, 90	Laser cutting		Smart laser cutting: OCT monitors laser ablation procedure
	Meyer C *et al*, 95	Other types of optical treatment		Some different methods based on thermal, photoacoustic, and thermomechanical effect of laser
Optical image- guided MIS	Wang C *et al*, 127	Optical molecular imaging guided surgery	Computer- and robot-assisted navigation, and autonomous robot for surgery	Optical biomarkers for detecting tumors and guiding surgery, benefits from safety, high-spatial resolution, and high-speed capability
	Shademan A* et al*, 128	Robot-assisted fluorescent surgery		First-time autonomous NIRF imaging guided robotic suturing
	Liu YY *et al*, 136	ICG-guided surgery		A better signal-to-background ratio (SBR), suffering from unclear tumor boundary
	Liu YY *et al*,^138^	Multimodal optical image-guided minimally-invasive surgery		Cross-scaling imaging, suffering from difficulty in registration of multimodal images
OCT-guided laser ablation	Fan Y *et al*, 140	OCT-guided laser ablation	Endoscopic theranostic catheter/platform, and real-time monitoring process of laser ablation	Common-scanning path integrated OCT and laser ablation
	Li Y *et al*, 144	OCT-guided automated laser ablation		Autonomous OCT guided laser ablation
	Katta N *et al*, 142,143,147	A fiber-laser platform		Common-path of OCT and ablation laser
	Yuan W *et al*, 150	Theranostic needle		Rigid probe integrated OCT and laser ablation
Fluorescence imaging-guided laser ablation	Liao H *et al*, 32,33	5-ALA fluorescence guided laser ablation	High-resolution and high-contrast imaging guided precise ablation; Endoscopic imaging with wide field of view (FOV) guided ablation	Preoperative MRI and intraoperative fluorescence guided autonomous robotic laser ablation at the first time
	Meyer T *et al*, 153	CARS-guided fs-ablation		A new CARS-guided fs-ablation platform
	Wang H *et al*, 154	Endomicroscopy imaging -guided ablation		Endoscopic imaging guided laser ablation (a surface-scanning device)
Multimodal imaging guidance of laser ablation	Murgu SD *et al*, 155	White light bronchoscopy (WLB), endobronchial ultrasound (EBUS), and OCT guided YAG ablation	Integration of multimodal imaging and laser using a single-port theranostic platform for treatment of cancers; Preoperative navigation and intraoperative multimodal imaging fusion guided ablation for minimally-invasive theranostics of cancers	Multimodal imaging (WLB, US, and OCT) guidance for laser ablation (Clinical research, case study)
	Nguyen VP *et al*, 156	PAM and OCT guided laser ablation		PAM and OCT guided laser ablation
	Basij M *et al*,^157^	Ultrasound and photoacoustic (PA)-guided laser ablation theranostic device		A platform and device integrated PAI guided laser ablation at the first time
	Yan Y *et al*, 159	An integrated PAI and HIFU guided laser and ultrasound		PAI guided laser and HIFU (*in vivo*)
OCT-guided PDT/PTT	Hamdoon Z *et al*, 167	OCT guided PDT	Endoscopic functional OCT guided PDT/PTT; Exploring the mechanism of PDT/PTT using functional OCT	Structural OCT image guided PDT
	Standish BA *et al*, 168	Doppler OCT guided PDT		Functional OCT guided PDT: Doppler, Angiography, and elastic imaging for exploring the mechanism of PDT and PTT
	Sirotkina MA *et al*, 169	OCTA guided PDT		
	Sirotkina MA *et al*, 171	OCTA and OCE guided PDT		
Multimodal optical image-guided PDT/PTT	Liu L *et al*, 174	NIRF/PA/CT multi-modality imaging guided PTT and PDT	Endoscopic functional optical imaging guided PDT/PTT; Exploring the mechanism of PDT/PTT using functional optical imaging	Based on tumor microenvironment- responsive nanocomposites
	Lee H *et al*, 8	Endoscopic bioelectronics and theranostic nanoparticles		An excellent MIT framework, suffers from the safety of theranostic nanoparticles, and the lack of endoscopic control strategies for MIT
	Sim C *et al*, 177	Photoacoustic imaging and near-infrared light-triggered theranostic platforms		Functional imaging to monitor theranostic effect, lack of effective theranostic method or devices
	Muhanna N* et al*, 180	Multimodal image-guided surgical and photodynamic interventions		Multimodal image-guided surgical and photodynamic interventions, lack of theranostic methods

## References

[1] Friedman AA, Letai A, Fisher DE, Flaherty KT (2015). Precision medicine for cancer with next-generation functional diagnostics. Nat Rev Cancer.

[2] Yun SH, Kwok SJJ (2017). Light in diagnosis, therapy and surgery. Nat Biomed Eng.

[3] Thekkek N, Richards-Kortum R (2008). Optical imaging for cervical cancer detection: solutions for a continuing global problem. Nat Rev Cancer.

[4] Johnson KW, Torres Soto J, Glicksberg BS, Shameer K, Miotto R, Ali M, Ashley E, Dudley JT (2018). Artificial Intelligence in Cardiology. J Am Coll Cardiol.

[5] Itatani Y, Obama K, Nishigori T, Ganeko R, Tsunoda S, Hosogi H, Hisamori S, Hashimoto K, Sakai Y (2019). Three-dimensional Stereoscopic Visualization Shortens Operative Time in Laparoscopic Gastrectomy for Gastric Cancer. Sci Rep.

[6] Degrauwe N, Hocquelet A, Digklia A, Schaefer N, Denys A, Duran R (2019). Theranostics in Interventional Oncology: Versatile Carriers for Diagnosis and Targeted Image-Guided Minimally Invasive Procedures. Front Pharmacol.

[7] Ai X, Mu J, Xing B (2016). Recent Advances of Light-Mediated Theranostics. Theranostics.

[8] Lee H, Lee Y, Song C, Cho HR, Ghaffari R, Choi TK (2015). An endoscope with integrated transparent bioelectronics and theranostic nanoparticles for colon cancer treatment. Nat Commun.

[9] Fuerst B, Wein W, Müller M, Navab N (2014). Automatic ultrasound-MRI registration for neurosurgery using the 2D and 3D LC(2) Metric. Med Image Anal.

[10] Pei P, Chen Y, Sun C, Fan Y, Yang Y, Liu X (2021). X-ray-activated persistent luminescence nanomaterials for NIR-II imaging. Nat Nanotechnol.

[11] Zausinger S, Schichor C, Uhl E, Reiser MF, Tonn J-C (2014). "Intraoperative CT in neurosurgery," in *Intraoperative Imaging and Image-Guided Therapy*, F. A. Jolesz, Ed. New York, NY: Springer New York.

[12] Pedro MT, Antoniadis G, Scheuerle A, Pham M, Wirtz CR, Koenig RW (2015). Intraoperative high-resolution ultrasound and contrast-enhanced ultrasound of peripheral nerve tumors and tumorlike lesions. Neurosurg Focus.

[13] Tang T, Azuma T, Iwahashi T, Takeuchi H, Kobayashi E, Sakuma I (2018). A high-precision US-guided robot-assisted HIFU treatment system for breast cancer. Engineering.

[14] Valdes PA, Bekelis K, Harris BT, Wilson BC, Leblond F, Kim A (2014). 5-Aminolevulinic acid-induced protoporphyrin IX fluorescence in meningioma: qualitative and quantitative measurements *in vivo*. Neurosurgery.

[15] Jermyn M, Mok K, Mercier J, Desroches J, Pichette J, Saint-Arnaud K (2015). Intraoperative brain cancer detection with Raman spectroscopy in humans. Sci Transl Med.

[16] Balog J, Sasi-Szabó L, Kinross J, Lewis MR, Muirhead LJ, Veselkov K (2013). Intraoperative tissue identification using rapid evaporative ionization mass spectrometry. Sci Transl Med.

[17] Li DY, Xia Q, Yu TT, Zhu JT, Zhu D (2021). Transmissive-detected laser speckle contrast imaging for blood flow monitoring in thick tissue: from Monte Carlo simulation to experimental demonstration. Light Sci Appl.

[18] Fink JR, Muzi M, Peck M, Krohn KA (2015). Multimodality Brain Tumor Imaging: MR Imaging, PET, and PET/MR Imaging. J Nucl Med.

[19] Chernomyrdin NV, Musina GR, Nikitin PV, Dolganova IN, Kucheryavenko AS, Alekseeva AI (2023). Terahertz technology in intraoperative neurodiagnostics: A review. Opto-Electron Adv.

[20] Ravi D, Fabelo H, Callic GM, Yang GZ (2017). Manifold Embedding and Semantic Segmentation for Intraoperative Guidance with Hyperspectral Brain Imaging. IEEE Trans Med Imaging.

[21] Leon R, Fabelo H, Ortega S, Piñeiro JF, Szolna A, Hernandez M (2021). VNIR-NIR hyperspectral imaging fusion targeting intraoperative brain cancer detection. Sci Rep.

[22] Fabelo H, Ortega S, Lazcano R, Madroñal D, M Callicó G, Juárez E (2018). An Intraoperative Visualization System Using Hyperspectral Imaging to Aid in Brain Tumor Delineation. Sensors (Basel).

[23] Wang LV, Hu S (2012). Photoacoustic tomography: *in vivo* imaging from organelles to organs. Science.

[24] Sun Y, Jiang H, O'Neill B (2011). Photoacoustic Imaging: An Emerging Optical Modality in Diagnostic and Theranostic Medicine. J Biosens Bioelectron.

[25] Yao J, Wang LV (2014). Photoacoustic Brain Imaging: from Microscopic to Macroscopic Scales. Neurophotonics.

[26] Chen Q, Qin W, Qi W, Xi L (2021). Progress of clinical translation of handheld and semi-handheld photoacoustic imaging. Photoacoustics.

[27] Kut C, Chaichana KL, Xi J, Raza SM, Ye X, McVeigh ER (2015). Detection of human brain cancer infiltration *ex vivo* and *in vivo* using quantitative optical coherence tomography. Sci Transl Med.

[28] Wang N, Lee CY, Park HC, Nauen DW, Chaichana KL, Quinones-Hinojosa A (2022). Deep learning-based optical coherence tomography image analysis of human brain cancer. Biomed Opt Express.

[29] Juarez-Chambi RM, Kut C, Rico-Jimenez JJ, Chaichana KL, Xi J, Campos-Delgado DU (2019). AI-Assisted *in situ* Detection of Human Glioma Infiltration Using a Novel Computational Method for Optical Coherence Tomography. Clin Cancer Res.

[30] Gunalan A, Mattos LS (2023). Towards OCT-Guided Endoscopic Laser Surgery-A Review. Diagnostics (Basel).

[31] Fillon M (2019). Surgery remains the best solution for patients with soft-tissue sarcomas. CA Cancer J Clin.

[32] Liao H, Shimaya K, Wang K, Maruyama T, Noguchi M, Muragaki Y (2008). Combination of intraoperative 5-aminolevulinic acid-induced fluorescence and 3-D MR imaging for guidance of robotic laser ablation for precision neurosurgery. Med Image Comput Comput Assist Interv.

[33] Liao H, Noguchi M, Maruyama T, Muragaki Y, Kobayashi E, Iseki H (2012). An integrated diagnosis and therapeutic system using intra-operative 5-aminolevulinic-acid-induced fluorescence guided robotic laser ablation for precision neurosurgery. Med Image Anal.

[34] Großerueschkamp F, Jütte H, Gerwert K, Tannapfel A (2021). Advances in Digital Pathology: From Artificial Intelligence to Label-Free Imaging. Visc Med.

[35] Liao H (2014). Integrated diagnostic and therapeutic techniques: Toward an intelligent medical system. Comput Med Imaging Graph.

[36] Waterhouse DJ, Fitzpatrick CRM, Pogue BW, O'Connor JPB, Bohndiek SE (2019). A roadmap for the clinical implementation of optical-imaging biomarkers. Nat Biomed Eng.

[37] Hollon T, Orringer DA (2021). Label-free brain tumor imaging using Raman-based methods. J Neurooncol.

[38] Huang D, Swanson EA, Lin CP, Schuman JS, Stinson WG, Chang W (1991). Optical coherence tomography. Science.

[39] Yang L, Chen Y, Ling S, Wang J, Wang G, Zhang B (2022). Research progress on the application of optical coherence tomography in the field of oncology. Front Oncol.

[40] Boppart SA, Brown JQ, Farah CS, Kho E, Marcu L, Saunders CM (2017). Label-free optical imaging technologies for rapid translation and use during intraoperative surgical and tumor margin assessment. J Biomed Opt.

[41] Fan Y, Xia Y, Zhang X, Sun Y, Tang J, Zhang L (2018). Optical coherence tomography for precision brain imaging, neurosurgical guidance and minimally invasive theranostics. Biosci Trends.

[42] Nathans J (2023). Seeing is believing: The development of optical coherence tomography. Proc Natl Acad Sci U S A.

[43] Wang J, Xu Y, Boppart SA (2017). Review of optical coherence tomography in oncology. J Biomed Opt.

[44] Vakoc BJ, Fukumura D, Jain RK, Bouma BE (2012). Cancer imaging by optical coherence tomography: preclinical progress and clinical potential. Nat Rev Cancer.

[45] Badhey AK, Schwarz JS, Laitman BM, Veremis BM, Westra WH, Yao M (2023). Intraoperative Use of Wide-Field Optical Coherence Tomography to Evaluate Tissue Microstructure in the Oral Cavity and Oropharynx. JAMA Otolaryngol Head Neck Surg.

[46] Gong P, Chin SL, Allen WM, Ballal H, Anstie JD, Chin L (2022). Quantitative Micro-Elastography Enables *In vivo* Detection of Residual Cancer in the Surgical Cavity during Breast-Conserving Surgery. Cancer Res.

[47] J (2022). Kang, R. Zhu, Y. Sun, J. Li, and K. Wong. Pencil-beam scanning catheter for intracoronary optical coherence tomography. Opto-Electronic Advances.

[48] Si P, Honkala A, de la Zerda A, Smith BR (2020). Optical Microscopy and Coherence Tomography of Cancer in Living Subjects. Trends Cancer.

[49] Rannen Triki A, Blaschko MB, Jung YM, Song S, Han HJ, Kim SI (2018). Intraoperative margin assessment of human breast tissue in optical coherence tomography images using deep neural networks. Comput Med Imaging Graph.

[50] Tampu IE, Eklund A, Johansson K, Gimm O, Haj-Hosseini N (2023). Diseased thyroid tissue classification in OCT images using deep learning: Towards surgical decision support. J Biophotonics.

[51] Y (2018). Fan, L. Ma, W. Chang, W. Jiang, S. Luo, X. Zhang, et al. Optimized optical coherence tomography imaging with hough transform-based fixed-pattern noise reduction. IEEE Access.

[52] Luo S, Fan Y, Chang W, Liao H, Kang H, Huo L (2019). Classification of human stomach cancer using morphological feature analysis from optical coherence tomography images. Laser Physics Letters.

[53] Chen PH, Lai HK, Yeh YC, Chang KW, Hou MC, Kuo WC (2022). En-face polarization-sensitive optical coherence tomography to characterize early-stage esophageal cancer and determine tumor margin. Biomed Opt Express.

[54] Mojahed D, Ha RS, Chang P, Gan Y, Yao X, Angelini B (2020). Fully Automated Postlumpectomy Breast Margin Assessment Utilizing Convolutional Neural Network Based Optical Coherence Tomography Image Classification Method. Acad Radiol.

[55] Goswami M (2021). Deep learning models for benign and malign ocular tumor growth estimation. Comput Med Imaging Graph.

[56] Yang Z, Soltanian-Zadeh S, Chu KK, Zhang H, Moussa L, Watts AE (2021). Connectivity-based deep learning approach for segmentation of the epithelium in *in vivo* human esophageal OCT images. Biomed Opt Express.

[57] Bareja R, Mojahed D, Hibshoosh H, Hendon C (2022). Classifying breast cancer in ultrahigh-resolution optical coherence tomography images using convolutional neural networks. Appl Opt.

[58] Zeng Y, Xu S, Chapman WC Jr LiS, Alipour Z, Abdelal H (2020). Real-time colorectal cancer diagnosis using PR-OCT with deep learning. Theranostics.

[59] Luo S, Ran Y, Liu L, Huang H, Tang X, Fan Y (2022). Classification of gastric cancerous tissues by a residual network based on optical coherence tomography images. Lasers Med Sci.

[60] Luo S, Fan Y, Liu H, An X, Xie H, Li P, Liao H, Huo L SVM based automatic classification of human stomach cancer with optical coherence tomography images. in *Conference on Lasers and Electro-Optics, OSA Technical Digest (online)* (Optica Publishing Group, 2018), paper JTu2A.99.

[61] Zhang J, Yuan W, Liang W, Yu S, Liang Y, Xu Z (2017). Automatic and robust segmentation of endoscopic OCT images and optical staining. Biomed Opt Express.

[62] Y (2020). Fan, S. Luo, L. Huo, Y. Liu, X. Li, H. Xie, et al. An imaging analysis and reconstruction method for multiple-micro-electro-mechanical system mirrors-based off-centre scanning optical coherence tomography probe. Laser Physics Letters.

[63] Luo S, Wang D, Tang J, Zhou L, Duan C, Wang D (2018). Circumferential-scanning endoscopic optical coherence tomography probe based on a circular array of six 2-axis MEMS mirrors. Biomed Opt Express.

[64] Pahlevaninezhad H, Khorasaninejad M, Huang YW, Shi Z, Hariri LP, Adams DC (2018). Nano-optic endoscope for high-resolution optical coherence tomography *in vivo*. Nat Photonics.

[65] Kirtane TS, Wagh MS (2014). Endoscopic Optical Coherence Tomography (OCT): Advances in Gastrointestinal Imaging. Gastroenterol Res Pract.

[66] Zulina N, Caravaca O, Liao G, Gravelyn S, Schmitt M, Badu K (2021). Colon phantoms with cancer lesions for endoscopic characterization with optical coherence tomography. Biomed Opt Express.

[67] Liu T, Pan T, Wang P, Qin S, Xie H (2022). Scanning optimization of an electrothermally-actuated MEMS mirror for applications in optical coherence tomography endoscopy. Sensors and Actuators A: Physical.

[68] Luo H, Li S, Zeng Y, Cheema H, Otegbeye E, Ahmed S (2022). Human colorectal cancer tissue assessment using optical coherence tomography catheter and deep learning. J Biophotonics.

[69] Otuya DO, Dechene NM, Poshtupaka D, Judson S, Carlson CJ, Zemlok SK (2022). Passively scanned, single-fiber optical coherence tomography probes for gastrointestinal devices. Lasers Surg Med.

[70] Lin L, Wang LV (2022). The emerging role of photoacoustic imaging in clinical oncology. Nat Rev Clin Oncol.

[71] Lin L, Hu P, Shi J, Appleton CM, Maslov K, Li L (2018). Single-breath-hold photoacoustic computed tomography of the breast. Nat Commun.

[72] Na S, Russin JJ, Lin L, Yuan X, Hu P, Jann KB (2022). Massively parallel functional photoacoustic computed tomography of the human brain. Nat Biomed Eng.

[73] Cao R, Nelson SD, Davis S, Liang Y, Luo Y, Zhang Y (2023). Label-free intraoperative histology of bone tissue via deep-learning-assisted ultraviolet photoacoustic microscopy. Nat Biomed Eng.

[74] Yang L, Li Y, Fang F, Li L, Yan Z, Zhang L (2021). Highly sensitive and miniature microfiber-based ultrasound sensor for photoacoustic tomography. Opto-Electronic Advances.

[75] Yang JM, Favazza C, Chen R, Yao J, Cai X, Maslov K (2012). Simultaneous functional photoacoustic and ultrasonic endoscopy of internal organs *in vivo*. Nat Med.

[76] Fornasarig M, Capuano A, Maiero S, Pivetta E, Guarnieri G, Canzonieri V (2021). pCLE highlights distinctive vascular patterns in early gastric cancer and in gastric diseases with high risk of malignant complications. Sci Rep.

[77] Jabbour JM, Saldua MA, Bixler JN, Maitland KC (2012). Confocal endomicroscopy: instrumentation and medical applications. Ann Biomed Eng.

[78] Zuo S, Hughes M, Yang GZ (2016). Novel Balloon Surface Scanning Device for Intraoperative Breast Endomicroscopy. Ann Biomed Eng.

[79] Spessotto P, Fornasarig M, Pivetta E, Maiero S, Magris R, Mongiat M (2017). Probe-based confocal laser endomicroscopy for *in vivo* evaluation of the tumor vasculature in gastric and rectal carcinomas. Sci Rep.

[80] Patel KB, Liang W, Casper MJ, Voleti V, Li W, Yagielski AJ (2022). High-speed light-sheet microscopy for the in-situ acquisition of volumetric histological images of living tissue. Nat Biomed Eng.

[81] Wang J, Li Y, Cao C, Zhou G, Li L (2021). High-fidelity and rapid cellular-level Mueller matrix imaging for tissue identification with unstained sections. Biomed Opt Express.

[82] Vahrmeijer AL, Hutteman M, van der Vorst JR, van de Velde CJ, Frangioni JV (2013). Image-guided cancer surgery using near-infrared fluorescence. Nat Rev Clin Oncol.

[83] Zhu M, Chang W, Jing L, Fan Y, Liang P, Zhang X (2019). Dual-modality optical diagnosis for precise *in vivo* identification of tumors in neurosurgery. Theranostics.

[84] Marques MJ, Hughes MR, Vyas K, Thrapp A, Zhang H, Bradu A (2019). En-face optical coherence tomography/fluorescence endomicroscopy for minimally invasive imaging using a robotic scanner. J Biomed Opt.

[85] Kong R, Dai C, Zhang Q, Gao L, Chen Z, Song Y (2022). Integrated US-OCT-NIRF Tri-Modality Endoscopic Imaging System for Pancreaticobiliary Duct Imaging. IEEE Trans Ultrason Ferroelectr Freq Control.

[86] Liu M, Chen Z, Zabihian B, Sinz C, Zhang E, Beard PC (2016). Combined multi-modal photoacoustic tomography, optical coherence tomography (OCT) and OCT angiography system with an articulated probe for *in vivo* human skin structure and vasculature imaging. Biomed Opt Express.

[87] Pahlevaninezhad H, Lee AM, Shaipanich T, Raizada R, Cahill L, Hohert G (2014). A high-efficiency fiber-based imaging system for co-registered autofluorescence and optical coherence tomography. Biomed Opt Express.

[88] Hoffman A, Manner H, Rey JW, Kiesslich R (2017). A guide to multimodal endoscopy imaging for gastrointestinal malignancy - an early indicator. Nat Rev Gastroenterol Hepatol.

[89] Jermakowicz WJ, Mahavadi AK, Cajigas I, Dan L, Guerra S, Farooq G (2019). Predictive modeling of brain tumor laser ablation dynamics. J Neurooncol.

[90] Jivraj J, Chen C, Huang Y, Ramjist J, Lu Y, Vuong B (2018). Smart laser osteotomy: integrating a pulsed 1064nm fiber laser into the sample arm of a fiber optic 1310nm OCT system for ablation monitoring. Biomed Opt Express.

[91] Mirza FN, Khatri KA (2017). The use of lasers in the treatment of skin cancer: A review. J Cosmet Laser Ther.

[92] Shapshay SM (1989). Laser technology in the diagnosis and treatment of head and neck cancer. Semin Surg Oncol.

[93] Colasanti R, Giannoni L, Dallari S, Liverotti V, Aiudi D, Di Rienzo A (2021). Application of a Scanner-Assisted Carbon Dioxide Laser System for Neurosurgery. World Neurosurg.

[94] Sartori S, Di Vece F, Ermili F, Tombesi P (2017). Laser ablation of liver tumors: An ancillary technique, or an alternative to radiofrequency and microwave?. World J Radiol.

[95] Meyer C, Bartsch D, Mirow N, Kirschbaum A (2017). Video-Assisted Laser Resection of Lung Metastases-Feasibility of a New Surgical Technique. Thorac Cardiovasc Surg.

[96] Quero G, Saccomandi P, Kwak JM, Dallemagne B, Costamagna G, Marescaux J (2019). Modular laser-based endoluminal ablation of the gastrointestinal tract: *in vivo* dose-effect evaluation and predictive numerical model. Surg Endosc.

[97] Rodríguez D, Sacco DE (2015). Minimally invasive surgical treatment for kidney stone disease. Adv Chronic Kidney Dis.

[98] Oguro Y (1994). Laser endoscopic treatment for early gastric cancer. J Gastroenterol.

[99] Fan Y, Xu L, Liu S, Li J, Xia J, Qin X The state-of-the-art and perspectives of laser ablation for tumor treatment. Cyborg and Bionic Systems.2023: n. pag.

[100] Jiang T, Chai W (2018). Endoscopic ultrasonography (EUS)-guided laser ablation (LA) of adrenal metastasis from pancreatic adenocarcinoma. Lasers Med Sci.

[101] Truong VG, Jeong S, Park JS, Tran VN, Kim SM, Lee DH (2021). Endoscopic ultrasound (EUS)-guided cylindrical interstitial laser ablation (CILA) on *in vivo* porcine pancreas. Biomed Opt Express.

[102] H (2020). Hareiza, D. Tan, K. Lim, Y. Chai, W. Yin, Abdullah, et al. A temperature-controlled laser hot needle with grating sensor for liver tissue tract ablation. IEEE Transactions on Instrumentation and Measurement.

[103] Korganbayev S, Orrico A, Bianchi L, Paloschi D, Wolf A, Dostovalov A (2021). PID controlling approach based on FBG array measurements for laser ablation of pancreatic tissues. IEEE Transactions on Instrumentation and Measurement.

[104] De Vita E, De Landro M, Massaroni C, Iadicicco A, Saccomandi P, Schena E (2021). Fiber Optic Sensors-Based Thermal Analysis of Perfusion-Mediated Tissue Cooling in Liver Undergoing Laser Ablation. IEEE Trans Biomed Eng.

[105] C (2021). Li and K. Wang. Effect of welding temperature and protein denaturation on strength of laser biological tissues welding. Optics & Laser Technology.

[106] Fan Y, Ma Q, Li M, Luan D, Kang H (2022). Quantitative investigation of laser ablation based on real-time temperature variations and OCT images for laser treatment applications. Lasers Surg Med.

[107] Di Matteo F, Martino M, Rea R, Pandolfi M, Panzera F, Stigliano E (2013). US-guided application of Nd:YAG laser in porcine pancreatic tissue: an *ex vivo* study and numerical simulation. Gastrointest Endosc.

[108] Schulmann N, Soltani-Sarvestani MA, De Landro M, Korganbayev S, Cotin S, Saccomandi P (2022). Model-Based Thermometry for Laser Ablation Procedure Using Kalman Filters and Sparse Temperature Measurements. IEEE Trans Biomed Eng.

[109] Marqa MF, Colin P, Nevoux P, Mordon SR, Betrouni N (2011). Focal laser ablation of prostate cancer: numerical simulation of temperature and damage distribution. Biomed Eng Online.

[110] Mohammadi A, Bianchi L, Korganbayev S, De Landro M, Saccomandi P (2022). Thermomechanical Modeling of Laser Ablation Therapy of Tumors: Sensitivity Analysis and Optimization of Influential Variables. IEEE Trans Biomed Eng.

[111] Franz P, Wang X, Zhu H, Chia R, Hasenberg T, Wang H (2020). Detection of blackbody radiation during fiber guided laser-tissue vaporization. Biomed Opt Express.

[112] Lipson RL, Baldes EJ, Olsen AM (1961). Hematoporphyrin derivative: a new aid for endoscopic detection of malignant disease. J Thorac Cardiovasc Surg.

[113] Triadafilopoulos G (2005). Photodynamic therapy for the treatment of patients with Barrett's high-grade dysplasia and mucosal adenocarcinoma. Nat Clin Pract Gastroenterol Hepatol.

[114] Dolmans DE, Fukumura D, Jain RK (2003). Photodynamic therapy for cancer. Nat Rev Cancer.

[115] Kachynski AV, Pliss A, Kuzmin AN, Ohulchanskyy TY, Baev A, Qu J (2014). Photodynamic therapy by *in situ* nonlinear photon conversion. Nature Photonics.

[116] He G, Li Y, Younis MR, Fu LH, He T, Lei S (2022). Synthetic biology-instructed transdermal microneedle patch for traceable photodynamic therapy. Nat Commun.

[117] Li J, Pumera M (2021). 3D printing of functional microrobots. Chem Soc Rev.

[118] Zhi D, Yang T, O'Hagan J, Zhang S, Donnelly RF (2020). Photothermal therapy. J Control Release.

[119] Li X, Lovell JF, Yoon J, Chen X (2020). Clinical development and potential of photothermal and photodynamic therapies for cancer. Nat Rev Clin Oncol.

[120] Zhen X, Pu K, Jiang X (2021). Photoacoustic Imaging and Photothermal Therapy of Semiconducting Polymer Nanoparticles: Signal Amplification and Second Near-Infrared Construction. Small.

[121] Arami H, Kananian S, Khalifehzadeh L, Patel CB, Chang E, Tanabe Y (2022). Remotely controlled near-infrared-triggered photothermal treatment of brain tumours in freely behaving mice using gold nanostars. Nat Nanotechnol.

[122] Xie Z, Peng M, Lu R, Meng X, Liang W, Li Z (2020). Black phosphorus-based photothermal therapy with aCD47-mediated immune checkpoint blockade for enhanced cancer immunotherapy. Light Sci Appl.

[123] York PA, Peña R, Kent D, Wood RJ (2021). Microrobotic laser steering for minimally invasive surgery. Sci Robot.

[124] Hu Y, Masamune K (2017). Flexible laser endoscope for minimally invasive photodynamic diagnosis (PDD) and therapy (PDT) toward efficient tumor removal. Opt Express.

[125] Keereweer S, Van Driel PB, Snoeks TJ, Kerrebijn JD, Baatenburg de Jong RJ, Vahrmeijer AL (2013). Optical image-guided cancer surgery: challenges and limitations. Clin Cancer Res.

[126] Sun Z, Jing L, Fan Y, Zhang H, Chen L, Wang G (2020). Fluorescein-guided surgery for spinal gliomas: Analysis of 220 consecutive cases. Int Rev Neurobiol.

[127] Wang C, Wang Z, Zhao T, Li Y, Huang G, Sumer BD (2018). Optical molecular imaging for tumor detection and image-guided surgery. Biomaterials.

[128] Shademan A, Decker RS, Opfermann JD, Leonard S, Krieger A, Kim PC (2016). Supervised autonomous robotic soft tissue surgery. Sci Transl Med.

[129] Zhong D, Chen W, Xia Z, Hu R, Qi Y, Zhou B (2021). Aggregation-induced emission luminogens for image-guided surgery in non-human primates. Nat Commun.

[130] Lee S, Namgoong JM, Kim Y, Cha J, Kim JK (2022). Multimodal Imaging of Laser Speckle Contrast Imaging Combined with Mosaic Filter-Based Hyperspectral Imaging for Precise Surgical Guidance. IEEE Trans Biomed Eng.

[131] Pshenay-Severin E, Bae H, Reichwald K, Matz G, Bierlich J, Kobelke J (2021). Multimodal nonlinear endomicroscopic imaging probe using a double-core double-clad fiber and focus-combining micro-optical concept. Light Sci Appl.

[132] Müller PC, Haslebacher C, Steinemann DC, Müller-Stich BP, Hackert T, Peterhans M (2021). Image-guided minimally invasive endopancreatic surgery using a computer-assisted navigation system. Surg Endosc.

[133] Catanzaro S, Copelli C, Manfuso A, Tewfik K, Pederneschi N, Cassano L (2017). Intraoperative navigation in complex head and neck resections: indications and limits. Int J Comput Assist Radiol Surg.

[134] Saeidi H, Ge J, Kam M, Opfermann JD, Leonard S, Joshi AS (2019). Supervised Autonomous Electrosurgery via Biocompatible Near-Infrared Tissue Tracking Techniques. IEEE Trans Med Robot Bionics.

[135] Kong SH, Haouchine N, Soares R, Klymchenko A, Andreiuk B, Marques B (2017). Robust augmented reality registration method for localization of solid organs' tumors using CT-derived virtual biomechanical model and fluorescent fiducials. Surg Endosc.

[136] Liu YY, Liao CH, Diana M, Wang SY, Kong SH, Yeh CN (2018). Near-infrared cholecystocholangiography with direct intragallbladder indocyanine green injection: preliminary clinical results. Surg Endosc.

[137] Diana M, Liu YY, Pop R, Kong SH, Legnèr A, Beaujeux R (2017). Superselective intra-arterial hepatic injection of indocyanine green (ICG) for fluorescence image-guided segmental positive staining: experimental proof of the concept. Surg Endosc.

[138] Liu YY, Kong SH, Diana M, Lègner A, Wu CC, Kameyama N (2016). Near-infrared cholecysto-cholangiography with indocyanine green may secure cholecystectomy in difficult clinical situations: proof of the concept in a porcine model. Surg Endosc.

[139] Boppart SA, Herrmann J, Pitris C, Stamper DL, Brezinski ME, Fujimoto JG (1999). High-resolution optical coherence tomography-guided laser ablation of surgical tissue. J Surg Res.

[140] Fan Y, Zhang B, Chang W, Zhang X, Liao H (2018). A novel integration of spectral-domain optical-coherence-tomography and laser-ablation system for precision treatment. Int J Comput Assist Radiol Surg.

[141] Li Z, Shen JH, Kozub JA, Prasad R, Lu P, Joos KM (2014). Miniature forward-imaging B-scan optical coherence tomography probe to guide real-time laser ablation. Lasers Surg Med.

[142] Katta N, McElroy AB, Estrada AD, Milner TE (2018). Optical coherence tomography image-guided smart laser knife for surgery. Lasers Surg Med.

[143] Katta N, Estrada AD, McElroy AB, Gruslova A, Oglesby M, Cabe AG (2019). Laser brain cancer surgery in a xenograft model guided by optical coherence tomography. Theranostics.

[144] Li Y, Fan Y, Hu C, Mao F, Zhang X, Liao H (2021). Intelligent optical diagnosis and treatment system for automated image-guided laser ablation of tumors. Int J Comput Assist Radiol Surg.

[145] Chang W, Fan Y, Zhang X, Liao H (2018). An Intelligent Theranostics Method Using Optical Coherence Tomography Guided Automatic Laser Ablation for Neurosurgery. Annu Int Conf IEEE Eng Med Biol Soc.

[146] Bayhaqi YA, Hamidi A, Canbaz F, Navarini AA, Cattin PC, Zam A (2022). Deep-Learning-Based Fast Optical Coherence Tomography (OCT) Image Denoising for Smart Laser Osteotomy. IEEE Trans Med Imaging.

[147] Katta N, Estrada AD, McErloy AB, Milner TE (2022). Fiber-laser platform for precision brain surgery. Biomed Opt Express.

[148] Maltais-Tariant R, Boudoux C, Uribe-Patarroyo N (2020). Real-time co-localized OCT surveillance of laser therapy using motion corrected speckle decorrelation. Biomed. Opt. Express.

[149] Kang J, Zhu R, Li J, Liu H, Ma X, Tao L (2021). Optical coherence tomography-surveilled laser ablation using multifunctional catheter and 355-nm optical pulses. Optics Communications.

[150] Yuan W, Chen D, Sarabia-Estrada R, Guerrero-Cázares H, Li D, Quiñones-Hinojosa A (2020). Theranostic OCT microneedle for fast ultrahigh-resolution deep-brain imaging and efficient laser ablation *in vivo*. Sci Adv.

[151] Tucker M, Ma G, Ross W, Buckland DM, Codd PJ (2021). Creation of an Automated Fluorescence Guided Tumor Ablation System. IEEE J Transl Eng Health Med.

[152] Lazarides AL, Whitley MJ, Strasfeld DB, Cardona DM, Ferrer JM, Mueller JL (2016). A Fluorescence-Guided Laser Ablation System for Removal of Residual Cancer in a Mouse Model of Soft Tissue Sarcoma. Theranostics.

[153] Meyer T, Ackermann R, Kammel R, Schmitt M, Nolte S, Tünnermann A (2019). CARS-imaging guidance for fs-laser ablation precision surgery. Analyst.

[154] Wang H, Ping Z, Fan Y, Kang H, Zuo S, A novel surface-scanning device for intraoperative tumor identification, therapy IEEE Access. 2019; 7: 96392-96403.

[155] Murgu SD, Colt HG, Mukai D, Brenner M (2010). Multimodal imaging guidance for laser ablation in tracheal stenosis. Laryngoscope.

[156] Nguyen VP, Li Y, Zhang W, Wang X, Paulus YM (2019). High-resolution multimodal photoacoustic microscopy and optical coherence tomography image-guided laser induced branch retinal vein occlusion in living rabbits. Sci Rep.

[157] Basij M, John S, Bustamante D, Kabbani L, Maskoun W, Mehrmohammadi M (2023). Integrated Ultrasound and Photoacoustic-Guided Laser Ablation Theranostic Endoscopic System. IEEE Trans Biomed Eng.

[158] Hazlewood D, Yang X (2019). Enhanced laser surface ablation with an integrated photoacoustic imaging and high intensity focused ultrasound system. Lasers Surg Med.

[159] Yan Y, John S, Shaik T, Patel B, Lam MT, Kabbani L (2021). Photoacoustic-guided endovenous laser ablation: Characterization and *in vivo* canine study. Photoacoustics.

[160] Tovar-Spinoza Z, Carter D, Ferrone D, Eksioglu Y, Huckins S (2013). The use of MRI-guided laser-induced thermal ablation for epilepsy. Childs Nerv Syst.

[161] Thompson SM, Callstrom MR, Knudsen BE, Anderson JL, Sutor SL, Butters KA (2013). Molecular bioluminescence imaging as a noninvasive tool for monitoring tumor growth and therapeutic response to MRI-guided laser ablation in a rat model of hepatocellular carcinoma. Invest Radiol.

[162] Fan Y, Sun Y, Chang W, Zhang X, Tang J, Zhang L (2018). Bioluminescence imaging and two-photon microscopy guided laser ablation of GBM decreases tumor burden. Theranostics.

[163] Chen Z, Milner TE, Wang X, Srinivas S, Nelson JS (1998). Optical Doppler tomography: imaging *in vivo* blood flow dynamics following pharmacological intervention and photodynamic therapy. Photochem Photobiol.

[164] Aalders MC, Triesscheijn M, Ruevekamp M, de Bruin M, Baas P, Faber DJ (2006). Doppler optical coherence tomography to monitor the effect of photodynamic therapy on tissue morphology and perfusion. J Biomed Opt.

[165] Liang CP, Nakajima T, Watanabe R, Sato K, Choyke PL, Chen Y (2014). Real-time monitoring of hemodynamic changes in tumor vessels during photoimmunotherapy using optical coherence tomography. J Biomed Opt.

[166] Chen D, Yuan W, Park HC, Li X (2020). *In vivo* assessment of vascular-targeted photodynamic therapy effects on tumor microvasculature using ultrahigh-resolution functional optical coherence tomography. Biomed Opt Express.

[167] Hamdoon Z, Jerjes W, Rashed D, Kawas S, Sattar AA, Samsudin R (2021). *In vivo* optical coherence tomography-guided photodynamic therapy for skin pre-cancer and cancer. Photodiagnosis Photodyn Ther.

[168] Standish BA, Yang VX, Munce NR, Wong Kee Song LM, Gardiner G, Lin A (2007). Doppler optical coherence tomography monitoring of microvascular tissue response during photodynamic therapy in an animal model of Barrett's esophagus. Gastrointest Endosc.

[169] Sirotkina MA, Matveev LA, Shirmanova MV, Zaitsev VY, Buyanova NL, Elagin VV (2017). Photodynamic therapy monitoring with optical coherence angiography. Sci Rep.

[170] Gubarkova EV, Feldchtein FI, Zagaynova EV, Gamayunov SV, Sirotkina MA, Sedova ES (2019). Optical coherence angiography for pre-treatment assessment and treatment monitoring following photodynamic therapy: a basal cell carcinoma patient study. Sci Rep.

[171] Sirotkina MA, Gubarkova EV, Plekhanov AA, Sovetsky AA, Elagin VV, Matveyev AL, Matveev LA (2020). *In vivo* assessment of functional and morphological alterations in tumors under treatment using OCT-angiography combined with OCT-elastography. Biomed Opt Express.

[172] Malhotra C, Shetty R, Kumar RS, Veluri H, Nagaraj H, Shetty KB (2012). *In vivo* imaging of riboflavin penetration during collagen cross-linking with hand-held spectral domain optical coherence tomography. J Refract Surg.

[173] Zhang X, Wang S, Cheng G, Yu P, Chang J (2022). Light-Responsive Nanomaterials for Cancer Therapy. Engineering.

[174] Liu L, Wang J, You Q, Sun Q, Song Y, Wang Y (2018). NIRF/PA/CT multi-modality imaging guided combined photothermal and photodynamic therapy based on tumor microenvironment-responsive nanocomposites. J Mater Chem B.

[175] Zhu M, Sheng Z, Jia Y, Hu D, Liu X, Xia X (2017). Indocyanine Green-holo-Transferrin Nanoassemblies for Tumor-Targeted Dual-Modal Imaging and Photothermal Therapy of Glioma. ACS Appl Mater Interfaces.

[176] Kadkhoda J, Tarighatnia A, Barar J, Aghanejad A, Davaran S (2022). Recent advances and trends in nanoparticles based photothermal and photodynamic therapy. Photodiagnosis Photodyn Ther.

[177] Sim C, Kim H, Moon H, Lee H, Chang JH, Kim H (2015). Photoacoustic-based nanomedicine for cancer diagnosis and therapy. J Control Release.

[178] Yue C, Zhang C, Alfranca G, Yang Y, Jiang X, Yang Y (2016). Near-Infrared Light Triggered ROS-activated Theranostic Platform based on Ce6-CPT-UCNPs for Simultaneous Fluorescence Imaging and Chemo-Photodynamic Combined Therapy. Theranostics.

[179] Wang L, Lu H, Gao Q, Yuan C, Ding F, Li J (2019). A multifunctional theranostic contrast agent for ultrasound/near infrared fluorescence imaging-based tumor diagnosis and ultrasound-triggered combined photothermal and gene therapy. Acta Biomater.

[180] Muhanna N, Cui L, Chan H, Burgess L, Jin CS, MacDonald TD (2016). Multimodal Image-Guided Surgical and Photodynamic Interventions in Head and Neck Cancer: From Primary Tumor to Metastatic Drainage. Clin Cancer Res.

[181] Wang X, Ramamurthy G, Shirke AA, Walker E, Mangadlao J, Wang Z (2020). Photodynamic Therapy Is an Effective Adjuvant Therapy for Image-Guided Surgery in Prostate Cancer. Cancer Res.

[182] Yin W, Pan F, Zhu J, Xu J, González-Rivas D, Okumura M Nanotechnology and nanomedicine: a promising avenue for lung cancer diagnosis and therapy. 2021;7(11): 9.

[183] Sano Y, Tanaka S, Kudo SE, Saito S, Matsuda T, Wada Y (2016). Narrow-band imaging (NBI) magnifying endoscopic classification of colorectal tumors proposed by the Japan NBI Expert Team. Dig Endosc.

[184] Gao T, Liu S, Wang A, Tang X, Fan Y (2023). Vascular elasticity measurement of the great saphenous vein based on optical coherence elastography. J Biophotonics.

[185] Lasker Foundation.

[186] Kim H, Youn S, Kim J, Park S, Lee M, Hwang J (2022). Deep laser microscopy using optical clearing by ultrasound-induced gas bubbles. Nature Photonics.

[187] Park J, Park B, Kim TY, Jung S, Choi WJ, Ahn J (2021). Quadruple ultrasound, photoacoustic, optical coherence, and fluorescence fusion imaging with a transparent ultrasound transducer. Proc Natl Acad Sci U S A.

[188] Ren W, Jiang J, Costanzo Mata AD, Kalyanov A, Ripoll J, Lindner S (2020). Multimodal imaging combining time-domain near-infrared optical tomography and continuous-wave fluorescence molecular tomography. Opt Express.

[189] Jacques SL (2013). Optical properties of biological tissues: a review [published correction appears in Phys Med Biol. 2013 Jul 21;58(14):5007-8]. Phys Med Biol.

[190] Smith AM, Mancini MC, Nie S (2009). Bioimaging: second window for *in vivo* imaging. Nat Nanotechnol.

[191] Zhang H, Liu X, Li Y, Wu W, Gu Y, Zhang T (2022). Study on the mechanism of thrombus ablation *in vitro* by burst-mode femtosecond laser. J Biophotonics.

[192] Dong L, Perrin N, Richer F, Roby-Brami A, Morel G (2022). The stability investigation of variable viscosity control in the human-robot interaction. Int J Med Robot.

[193] Keller B, Draelos M, Zhou K, Qian R, Kuo A, Konidaris G (2020). Optical Coherence Tomography-Guided Robotic Ophthalmic Microsurgery via Reinforcement Learning from Demonstration. IEEE Trans Robot.

[194] Saeidi H, Opfermann JD, Kam M, Wei S, Leonard S, Hsieh MH (2022). Autonomous robotic laparoscopic surgery for intestinal anastomosis. Sci Robot.

[195] Hacker L, Wabnitz H, Pifferi A, Pfefer TJ, Pogue BW, Bohndiek SE (2022). Criteria for the design of tissue-mimicking phantoms for the standardization of biophotonic instrumentation. Nat Biomed Eng.

[196] Amarnani R, Shende P (2021). Microneedles in diagnostic, treatment and theranostics: An advancement in minimally-invasive delivery system. Biomed Microdevices.

[197] Omisore OM, Han S, Xiong J, Li H, Li Z, Wang L (2020). A review on flexible robotic systems for minimally invasive surgery. IEEE Transactions on Systems, Man, and Cybernetics: Systems.

[198] Nassani N, Alsheikh M, Carroll B, Nguyen D, Carroll RE (2020). Theranostic Gastrointestinal Endoscopy: Bringing Healing Light to the Lumen. Clin Transl Gastroenterol.

[199] Zuo S, Yang GZ (2017). Endomicroscopy for Computer and Robot Assisted Intervention. IEEE Rev Biomed Eng.

[200] Wang K, Chi C, Hu Z, Liu M, Hui H, Shang W, at al (2015). Optical Molecular Imaging Frontiers in Oncology: The Pursuit of Accuracy and Sensitivity. Engineering.

